# Lysolipids are prominent in subretinal drusenoid deposits, a high-risk phenotype in age-related macular degeneration

**DOI:** 10.3389/fopht.2023.1258734

**Published:** 2023-11-24

**Authors:** David M. G. Anderson, Ankita Kotnala, Lukasz G. Migas, N. Heath Patterson, Léonore E. M. Tideman, Dongfeng Cao, Bibek Adhikari, Jeffrey D. Messinger, Thomas Ach, Sara Tortorella, Raf Van de Plas, Christine A. Curcio, Kevin L. Schey

**Affiliations:** ^1^ Department of Biochemistry, Vanderbilt University, Nashville, TN, United States; ^2^ Department of Ophthalmology and Visual Sciences, University of Alabama at Birmingham, Birmingham, AL, United States; ^3^ Delft Center for Systems and Control (DCSC), Delft University of Technology, Delft, Netherlands; ^4^ Vision Science Graduate Program, University of Alabama at Birmingham, Birmingham, AL, United States; ^5^ Department of Ophthalmology, University Hospital Bonn, Bonn, Germany; ^6^ Molecular Horizon Srl, Perugia, Italy

**Keywords:** age-related macular degeneration, imaging mass spectrometry, subretinal drusenoid deposit, retinal pigment epithelium, lysolipid, interpretable supervised machine learning, SHAP maps

## Abstract

**Introduction:**

Age related macular degeneration (AMD) causes legal blindness worldwide, with few therapeutic targets in early disease and no treatments for 80% of cases. Extracellular deposits, including drusen and subretinal drusenoid deposits (SDD; also called reticular pseudodrusen), disrupt cone and rod photoreceptor functions and strongly confer risk for advanced disease. Due to the differential cholesterol composition of drusen and SDD, lipid transfer and cycling between photoreceptors and support cells are candidate dysregulated pathways leading to deposit formation. The current study explores this hypothesis through a comprehensive lipid compositional analysis of SDD.

**Methods:**

Histology and transmission electron microscopy were used to characterize the morphology of SDD. Highly sensitive tools of imaging mass spectrometry (IMS) and nano liquid chromatography tandem mass spectrometry (nLC-MS/MS) in positive and negative ion modes were used to spatially map and identify SDD lipids, respectively. An interpretable supervised machine learning approach was utilized to compare the lipid composition of SDD to regions of uninvolved retina across 1873 IMS features and to automatically discern candidate markers for SDD. Immunohistochemistry (IHC) was used to localize secretory phospholipase A2 group 5 (PLA2G5).

**Results:**

Among the 1873 detected features in IMS data, three lipid classes, including lysophosphatidylcholine (LysoPC), lysophosphatidylethanolamine (LysoPE) and lysophosphatidic acid (LysoPA) were observed nearly exclusively in SDD while presumed precursors, including phosphatidylcholine (PC), phosphatidylethanolamine (PE) and phosphatidic acid (PA) lipids were detected in SDD and adjacent photoreceptor outer segments. Molecular signals specific to SDD were found in central retina and elsewhere. IHC results indicated abundant PLA2G5 in photoreceptors and retinal pigment epithelium (RPE).

**Discussion:**

The abundance of lysolipids in SDD implicates lipid remodeling or degradation in deposit formation, consistent with ultrastructural evidence of electron dense lipid-containing structures distinct from photoreceptor outer segment disks and immunolocalization of secretory PLA2G5 in photoreceptors and RPE. Further studies are required to understand the role of lipid signals observed in and around SDD.

## Introduction

Age related macular degeneration (AMD) causes legal blindness worldwide and affects more individuals as populations age (1). Neovascular complications of AMD are managed medically. An inhibitor of a complement protein was recently approved to slow the expansion of atrophy (2, 3), i.e., loss of photoreceptors and supporting retinal pigment epithelium (RPE). However, the lack of targeted treatments and preventions for the vast majority of AMD patients with early-stage disease prompts continued molecular investigation for new insight.

Fifteen years of managing neovascular AMD using optical coherence tomography (OCT) to visualize retinal layers has led to the concept of deposit-driven disease (4). Distinct forms of neovascularization and atrophy are preceded by accumulation and expansion of pathologic extracellular deposits on either side of the RPE. Drusen are located between the RPE basal lamina (RPE-BL) and inner collagenous layer (ICL) of Bruch’s membrane (BrM) (5, 6) and are most prominent under the cone-only fovea (7). In contrast, subretinal drusenoid deposits (SDD; also called reticular pseudodrusen) are located between photoreceptors and the RPE and first appear in the rod-rich perifovea and near-periphery (8, 9).

SDD are poorly visualized on color fundus photography and thus their importance in AMD progression received attention only within the OCT era (4). Depending on the patient population and imaging technology, 30-40% of eyes with intermediate AMD may have SDD (10). SDD constitute an independent risk factor for progression especially for neovascularization originating from the retinal vasculature and are present in most eyes with geographic atrophy (4). Due to the differential cholesterol composition of drusen and SDD, lipid transfer and cycling between photoreceptors and support cells are candidate dysregulated pathways (11, 12). Due to the topographic similarity of drusen to foveal cone-mediated vision and SDD to rods, these pathways may represent constitutive functions specific to each class of photoreceptors. Prominent aging changes in choriocapillaris microvasculature and Bruch’s membrane (“floor of microangiopathy”), due to a lifetime of serving metabolically active outer retina, block outwardly flowing metabolites and wastes and contribute to deposit formation in persons at genetic risk (13–16).

Matrix-assisted laser desorption ionization mass spectrometry imaging (MALDI IMS) is a powerful analytical technique to visualize analyte (lipid, protein, or metabolite) abundance across a tissue section. MALDI IMS has elucidated tissue constituents from eyes of laboratory animals (17–26) and human donors (27, 28). Using lipid-specific matrices, MALDI IMS localizes lipids while maintaining tissue topography, making it ideal to study pathology involving lipids in specific locations in AMD retinas (9, 11, 12). We previously showed that many lipid species vary in signal intensity across retinal regions and between neurosensory retina and supporting RPE-choroid. These previously unrecognized specificities in lipid localization and contributory pathways include activities related to lipophilic vitamin A, needed for phototransduction (23, 28).

Although lipid homeostasis is crucial to RPE and photoreceptor health (29), mechanisms related to SDD biogenesis are largely unknown. The current study comprehensively analyzes the composition of human SDD using MALDI IMS and nano liquid chromatography tandem mass spectrometry (nLC-MS/MS) (28, 30). To further inform on the mechanism of deposit formation, an interpretable supervised machine learning approach was utilized to compare SDD tissue regions to regions of uninvolved retina, and to automatically discern potential SDD marker candidates among hundreds of IMS-mapped molecular species. This analysis is supplemented with ultrastructure examination of SDD at different clinical stages to account for membranes of adjacent cells and subcellular features containing lipids and immunohistochemical localization of a mechanistic candidate phospholipase.

## Materials and methods

Additional experimental details are in **Supplementary Materials**. An overview of the workflow is shown in **Figure S1**.

### Tissue collection

Tissues were prepared as described by Anderson et al. (28). Whole eyes were obtained from deceased human donors by Advancing Sight Network (Birmingham, AL) as part of ongoing studies on age-related macular degeneration (AMD) that are approved by institutional review at University of Alabama at Birmingham (protocol # N170213002), where tissues were collected. In total, 8 donor eyes were used in this study and details of each donor and how the eyes were used are included in **Table S1**. With the exception of the eyes used for immunohistochemistry, all eyes had early or atrophic AMD, but not neovascular AMD. For IMS studies, whole globes were opened anteriorly and immersed in 4% phosphate-buffered paraformaldehyde (PFA) overnight. Globes were then placed in 1% PFA at 4°C for up to 48 h prior to sectioning. Dissected tissue containing central retina with the fovea was embedded in 2.25% carboxymethylcellulose (CMC) before sectioning at 12 µm thickness and mounting onto indium tin oxide (ITO) coated microscope slides (Delta Technologies ETC). Samples were vacuum sealed with oxygen absorbing packets and transported to Vanderbilt University on dry ice and stored in a -80°C freezer. Before analysis, slides were brought to room temperature and dried in a vacuum desiccator for a minimum of 30 minutes.

### MALDI IMS analysis

MALDI matrices, 1,5-diaminonaphthalene (DAN) for negative ion mode and 2,5-dihydroxyacetophenone (DHA) for positive ion mode, were applied to tissue sections using a custom designed sublimation device. MALDI IMS data were acquired with a 10 μm pixel size with a 10 µm pitch in full scan mode using a timsTOF Pro MALDI imaging platform in QTOF mode (Bruker Daltonik, Bremen, Germany). Data were acquired with 250 laser shots per pixel and within a mass range of m/z 300−2000. The mass spectrometer was calibrated with red phosphorus prior to data acquisition (31). Following IMS data acquisition, the same tissue sections were stained with hematoxylin and eosin (H&E) to permit spatial registration of IMS signals to tissue layers. Sections were imaged by bright-field microscopy and, both pre- and post-IMS acquisition, by fluorescence microscopy for autofluorescence (AF), which is especially intense for the RPE layer. For H&E staining, the matrix was removed with a light 100% methanol rinse. Slides were immersed in 95 and 70% ethanol for 30 seconds before a 20 second dip in distilled H_2_O, slides were then placed in hematoxylin for 3 minutes then rinsed by sequential dips (~5) in clean H_2_O. Tissues were subsequently dehydrated by immersing slides in 70 and 95% ethanol for 30 seconds, followed by 1 minute in eosin. Excess eosin was removed by immersing the slides in 95 and 100% ethanol for 30 seconds each before placing the slide in xylene for 3 minutes and mounting the coverslip with (Cytoseal XYL, ThermoFisher Scientific). Data were initially processed using SCiLS Lab MVS (Version 2023a Pro) to find ions of interest before exporting the data to an advanced image registration workflow using *IMS MicroLink* and *wsireg*, incorporating both AF and bright-field microscopy images in the Vitessce visualization tool (28, 32–34). Images were thresholded on an individual basis to give the best contrast to and to allow for clear localizations to be determined. IMS data were exported for accurate registration as TIFF images.

### MALDI IMS data processing

Data were exported from the Bruker timsTOF file format (.d) to a custom binary format for ease of access and improved performance. Each pixel/frame contains between 10^4^-10^5^ centroid peaks that cover the entire acquired m/z range, which can be used to reconstitute a pseudo-profile mass spectrum using Bruker’s timsTOF SDK software (v2.21). The IMS datasets were m/z-aligned using internal peaks, specifically, 6 peaks that appeared in at least 50% of the pixels, using the *msalign* library (v0.2.0) (35). This step corrects spectral misalignments (drift along the m/z domain), resulting in improved overlap between spectral features (peaks) across the group of IMS datasets. Subsequently, the mass axis of each dataset was calibrated using a minimum of 4 calibrant species to correct for mass errors, where each dataset was corrected to approximately ±1 ppm precision. After m/z alignment and calibration, an average mass spectrum for each dataset was computed across all pixels of that IMS dataset, specific to each ionization mode. The aim was feature detection in the average mass spectra from all IMS datasets in each ionization mode; however, despite the alignment and calibration, the mass axes are still slightly different for each dataset. To overcome this, we resample each mass spectrum in each dataset to a single common mass axis with slightly rougher spectral sampling (approximately 1.5 ppm; spacing between adjacent mass bins) and only then calculate an average mass spectrum. For each ionization mode, the average mass spectrum was peak-picked and a total of 983 and 890 peaks were detected in the positive and negative ionization mode, respectively. Note that isotopic peaks were not removed in the classification workflow. Following these m/z-axis pre-processing steps, we computed normalization correction factors for each of the pixels in the datasets to assure comparability of ion intensities across IMS datasets. To this end, we used a total ion current (TIC) strategy, modified for robustness, where only intensity data between the 5th and 95th percentiles is summed together to form the TIC (5-95th TIC). The intensity values that fall outside of this 5/95th percentile window were exempted from the normalization correction to reduce the impact of potential outlier peaks/features on normalization.

### Supervised machine learning analysis and automated marker candidate discovery

To avoid subjective assessment and to ensure an exhaustive search for SDD-relevant marker candidates among the hundreds of ions mapped by IMS, we employed an interpretable supervised machine learning approach to compare SDD to regions of uninvolved retina and to automatically discern candidate markers. A complete description of the approach can be found in Tideman et al. (36). Briefly, we first trained a multivariate classification model that assigns IMS pixels to different biological classes, i.e., “SDD tissue area” versus “non-SDD tissue area”, based on the mass spectrum of each pixel. Subsequently, an interpretable machine learning approach named Shapley additive explanations (SHAP) was used to examine how the trained classification model made decisions to label IMS pixels as ‘SDD’ or ‘non-SDD’. The SHAP method allowed all IMS-measured molecular species to be automatically ranked in decreasing order of relevance to the model’s recognition of SDD. The top-ranked ion species on this list are marker candidates for SDD, either by positive or negative correlation to SDD.

### Data labelling/mask creation

To make the classification model capture IMS variation that is relevant to retina tissue with SDD, we labelled IMS pixels as either *SDD* or *background*. The positive class (pixels labeled as *SDD*) was manually assigned in napari (v0.4.2) (37), based on low-dimensional latent patterns extracted by non-negative matrix factorization of the IMS data. Our initial labeling was subsequently compared to features seen by histological staining and corrected if needed. The negative class (*background*-labeled pixels) was automatically created sampling non-positive class pixels, effectively excluding SDD pixels from being in the *background* class. Since the number of background-labeled pixels is much larger than the number of *SDD*-labeled pixels, we performed auto-balancing of the training data. The balancing is performed by randomly sampling examples from the negative class pixels such that the number of pixels in the positive and negative class is roughly equal (~5,700 pixels/class in negative ion mode and ~3,650 pixels/class in positive ion mode).

### Building an SDD classification model

We employed eXtreme Gradient Boosting (XGBoost) (38) to learn a tree-based model that can classify mass spectra as either *SDD* or *background*. The model was trained with a 67%/33% train/test split. The classification workflow was carried out using the scikit-learn (v1.0.2) and XGBoost (v1.5.2) libraries in Python (v3.8.11). The classifier models achieved good SDD-recognition performance for negative and positive ion mode (balanced accuracy, 0.9854, 0.9821; precision 0.9911, 0.9831; recall 0.9798, 0.9815). These numbers indicate that, for both ionization modes, a model was found that can recognize SDD well given the IMS-detected ions provided. Since these models capture a strong connection between the IMS data and SDD, exploring their decision process by SHAP can yield molecular species relevant to SDD.

### Lipid extraction and nLC-MS/MS analysis

Sections of two human donor retinas with SDD were processed for lipid extraction for nLC-MS/MS analysis (**Table S1**). Twelve-μm-thick paraformaldehyde-fixed tissue sections were extracted. Under a dissecting microscope, sclera was removed with a number 10 scalpel blade. From the remaining retina-choroid tissue, regions with SDD were scraped from the glass slide and placed in a 1.5-mL HPLC glass vial. One milliliter of MMC extraction solvent (1.3:1:1, methanol: methyl tert-butyl ether: chloroform) was spiked with 10 μL of lipid standard mixture (SPLASH® LIPIDOMIX® Mass Spec Standard, Avanti Polar Lipids, Alabaster, AL, USA), vortexed for 60 s and centrifuged at 3000 rpm for 10 min (30, 39). The supernatant was transferred to a separate HPLC vial and was evaporated to dryness. The sample was then reconstituted with 10 μL n-butanol: isopropyl aclcohol:H_2_0 (8:23:69) with 5 mM phosphoric acid, and 1 μL was injected each for positive and negative ion mode analysis by nLC–MS/MS.

In-house packed reverse phase columns (20 cm x 75 µm) were packed with 1.9 µm BEH C-18 material and nLC-MS/MS analysis was performed using an EASY nLC 1000 (Thermo Scientific). A gradient mobile phase comprised of solvent A (10 mM ammonium formate in 40:60 water: acetonitrile by volume with 0.1% formic acid) and Solvent B (10 mM ammonium formate in 90:10 isopropanol: acetonitrile by volume with 0.1% formic acid) was used. A 300 nL/min flow rate was used for 120 minutes, and the column compartment was heated to 60°C. The gradient elution profile was as follows: 1-30% B (0-12 min), 30%–51% B (12-16 min), 51% B (16-20 min), 51%–61% B (20–40min), 61%–71% B (40-60 min), 71%–99% B (60–80 min), 99% B (80–100 min), 99%–1% B (100–110 min), 1% B (110–120 min) (40, 41).

HPLC eluate flowed into an ESI source, and ions were analyzed using a Q Exactive HF instrument (Thermo Scientific, San Jose, CA, USA). Data were acquired in both full (MS1) and data dependent MS2 (ddMS2) scan modes using positive and negative modes separately. The full scan mode had a mass resolution of 60,000, a mass range of m/z 200–2000, and a maximum trap fill time of 100 ms. ddMS2 data were acquired at 15,000 resolution, with a maximum trap fill time of 160 ms. The isolation window of selected MS1 ions was ±1.4 m/z with a normalized collision energy (NCE) of 20 and 25. LC–MS/MS data were acquired using Xcalibur version 4.0.

### Lipid identification

Both positive and negative mode MALDI timsTOF raw data files were imported into LipostarMSI software (Molecular Horizon srl, Perugia, IT) (42) for processing, image co-registration, statistical analysis, and lipid identification. Data were recalibrated using the m/z 885.5499 signal in negative ion mode and m/z 725.5456, m/z 756.5514, m/z 760.5851, m/z 782.5670, and m/z 784.5851 in positive ion mode. nLC–MS/MS data acquired from serial section homogenates were imported into LipostarMSI to identify lipids in the SDD regions of interest (ROIs). ROIs were determined based on histology and on supervised data analysis [PCA and bisecting k-means segmentation (43)]. A co-localization algorithm was then applied to isolate the ion markers statistically correlated to the pathological ROIs, further confirmed by visual inspection of ion images (44). Molecular identification was automatically assigned by LipostarMSI based on accurate MS and MS/MS matching (≤10 ppm, possible adducts: +H, +Na, +K, -H, +Cl; dimers and oxidized species for GP and GL allowed) against the LIPIDMAPS Database (http://www.lipidmaps.org/ accessed on 6 January 2022). Only molecules that could be definitively identified by LC-MS/MS fragments were reported. The entire data analysis workflow was performed independently on two biological replicates.

### PASH staining and light microscopy

For AMD diagnosis and comparison to *ex vivo* OCT, cryosections through the fovea and perifovea (recognized by Henle fiber layer) near those used for MALDI-IMS were stained with periodic acid Schiff hematoxylin (PASH, Poly Scientific R&D Corp., Bay Shore, NY, USA; #K047 kit). This stain highlights Bruch’s membrane and sub-RPE deposits. Sections retained from a previous immunohistochemistry study (45), with overall better morphologic preservation, were also perused for good examples of SDD. Slides were dehydrated through 85%, 95%, 100% ethanol, and 100% xylene (Fisher, # X3S-4) for 5 minutes, twice at each concentration. All slides were cover-slipped with permanent medium (Permount, EMS, # 17986-01) and air-dried in a hood overnight.

To facilitate translation to clinical OCT imaging, which has signal in every pixel, bright field images were acquired for all stained slides. One stained section per slide was scanned with a 20X objective and a robotic microscope stage (BX61VS, VSI 120, CellSens; Olympus, Center Valley PA), scaled to tissue units. Sections were centered on the fovea or for non-foveal sections, where Henle fibers diverge, using a custom plugin for FIJI (https://imagej.nih.gov/ij/download.html). *Ex vivo* OCT B-scans and scanned whole sections were matched for major landmarks (e.g., overall tissue contour, foveal center, large vessels, individual pathologies). For details, some stained sections were scanned with a 60X oil immersion objective (numerical aperture = 1.4).

We used differential interference contrast (DIC) microscopy with 40X and 60X objectives to determine if material was visible in unstained dome- or flame-shaped areas lacking photoreceptor outer segments in the subretinal space. To compare our tissue sample to a clinical OCT staging system, we assessed SDD height relative to photoreceptors in cryosections stained for PASH in these and other similarly accessioned and processed AMD eyes (8, 45, 46). Stage 1 deposits impacted only outer segments, Stage 2 reached the inner segments, and Stage 3 deposits intruded on inner segments.

### Transmission electron microscopy

To visualize the ultrastructure of SDD and membranes of cells surrounding the deposits, we prepared AMD tissue samples from prior studies for transmission electron microscopy. One block (Case 1, 76-year-old female) came from a histologic study of micro-dissected and pelleted drusen (47). Another block (Case 2, 88-year-old-male) came from macula-wide sections processed for the Project MACULA website (48). Both were preserved in 1% glutaraldehyde and 2.5% paraformaldehyde in phosphate buffer. To preserve extracellular lipids, samples were post-fixed with osmium tannic acid paraphenylenediamine (OTAP) (49, 50). Tissue blocks from Cases 1-2 were embedded in epoxy resin (PolyBed 812, EMS, Hatfield PA) and re-sectioned for this study. Gold sections (nominally 90 nm thick) were mounted on grids (Formvar Carbon Support Film on Specimen Grid, Electron Microscopy Sciences, Fort Washington, PA) and post-stained with mixed lanthanides (UranyLess, Electron Microscopy Sciences, Fort Washington, PA). TEM images were acquired at original magnifications of up to 2100x (Tecnai 120kv TEM, FEI, Hillsboro, OR; BioSprint 29 Megapixel CCD camera, AMT, Woburn, MA). An electron micrograph of clinically documented SDD (Case 3) was made available from the Sarks Archive (Sydney Australia; Data Use Agreement 2021STE02369); this eye had been post-fixed in 2% osmium.

### Immunohistochemistry

Our finding of abundant lysolipids in SDD, and the finding that the monogenic inherited disorder phospholipase A2 group V (PLA2G5) retinopathy has an SDD phenotype (27, 28), prompted us to confirm that the gene product localized to the retina. Cryosections from two normal donor eyes were processed for colorimetric immunohistochemical staining with rabbit polyclonal anti-human PLA2G5 (Fisher Scientific, Cat# PIPA584150, https://www.fishersci.com/shop/products/pla2g5polyclonal-antibody-invitrogen-1/PIPA584150). Briefly, after heat-induced antigen retrieval with unmasking solution (Vector Labs, Cat# H-3300) and 3 blocking steps (BLOXALL, Vector labs, Cat# SP-6000), avidin/biotin blocking kit (Vector Labs, Cat# SP-2001) and 10% horse serum (Vector Labs, Cat# S2000-20) in 1x blocking solution (ThermoFisher, Cat# PI37525), cryosections were incubated with PLA2G5 primary antibody (1: 50, ThermoFisher, Cat#PA584150), horse antirabbit biotinylated secondary antibody, ABC Complex solution (Vector Labs, Cat# PK-7200), and diaminobenzidine peroxidase substrate (Vector Labs, Cat# SK-4100) for the desired brown color. Stained sections on glass slides were scanned with a 20X, 40X, and 60X objectives (0.75, 0.95, and 1.42 numerical aperture, respectively) and a robotic microscope stage (Olympus VS120, Olympus Japan).

### Preparation of microscopy figures

Images for figures were assembled and adjusted for contrast, exposure, and sharpness to maximize the intensity histogram for contrast and white balance (Photoshop, CS6; Adobe Systems, San Jose, CA). To facilitate translation of results to clinical OCT, retinal layers were horizontally oriented with choroid down in all figures.

## Results

The goal of this study was to combine morphological, ultrastructural, and molecular analysis of SDD to characterize deposit lipid composition and, with the aid of IMS and interpretable supervised machine learning, to propose candidate sources for these molecules.

### Recognizing SDD in donor eyes

**Figure 1:**
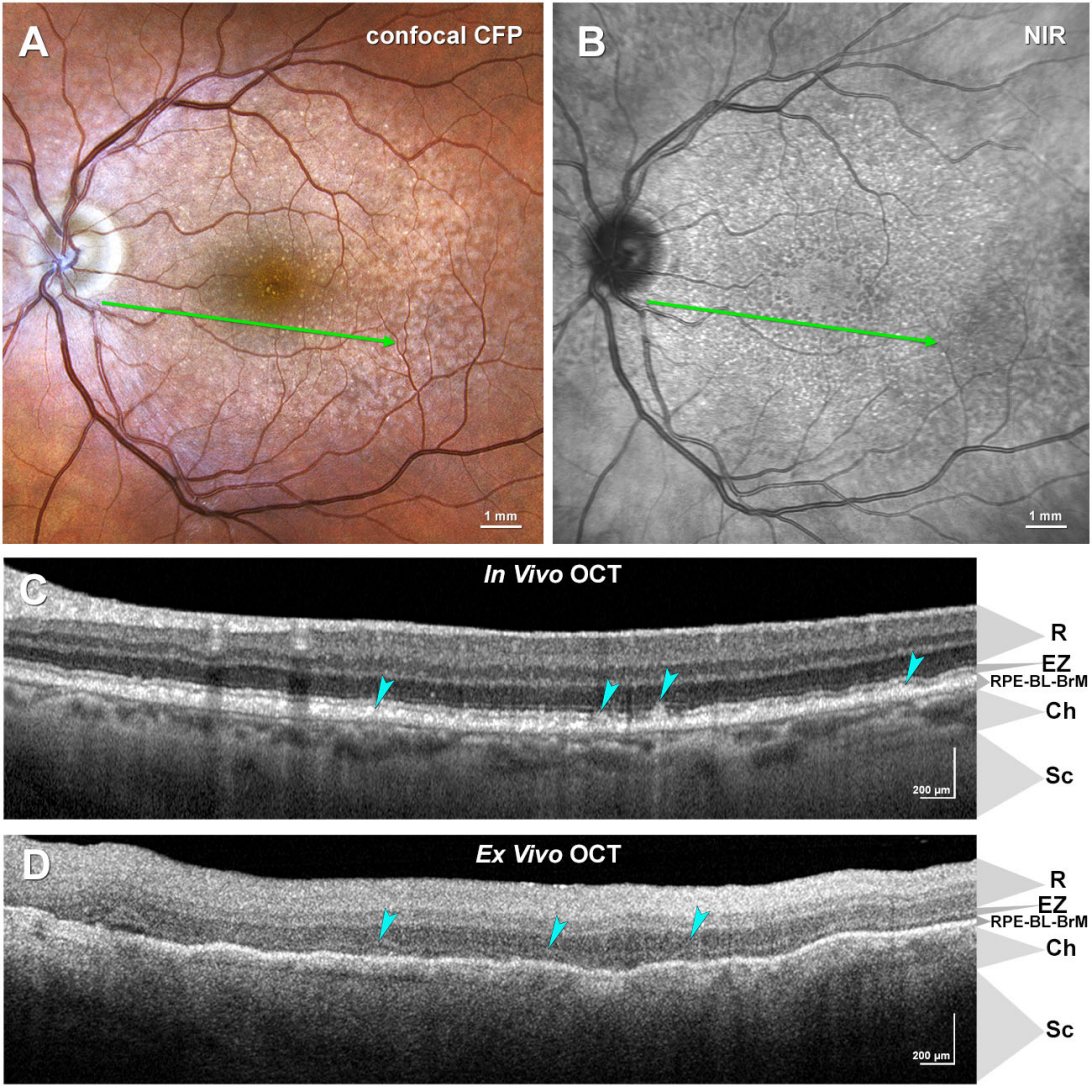
Multimodal imaging of subretinal drusenoid deposits (SDD) in age-related macular degeneration (AMD). **(A, B)** Confocal color fundus photograph (CFP), **(A)** and near-infrared reflectance (NIR), **(B)** show SDD in an AMD patient as yellow dots and hyporeflective spots, respectively. The green arrow indicates the location of the *in vivo* optical coherence tomography (OCT) B-scan image in **(C)**. **(C)** *In vivo* OCT B-scan exhibits SDD lesions between external limiting membrane (ELM) and retinal pigment epithelium (RPE), as indicated by cyan arrowheads. **(D)** *Ex vivo* OCT B-scan of a donor eye with AMD (not the same eye as in **(A–C)**) reveals SDD above the RPE band. Ch, choroid; EZ, ellipsoid zone; R, retina; RPE-BL-BrM, RPE-basal lamina-Bruch’s membrane band; Sc, Sclera. Clinical images courtesy of **(D)** Kar, M.E. Clark, and **(C)** Owsley (NIH R01EY029595).

**Figures 1A, B** shows that in clinical color fundus photography and near-infrared reflectance imaging of a representative intermediate AMD eye, SDD appears as regularly spaced yellow dots and hyporeflective spots, respectively. By clinical OCT (**Figure 1C**), SDD lesions appear as regularly spaced reflective mounds between the external limiting membrane (ELM) and RPE-basal lamina-Bruch’s membrane band. The largest deposits (stage 3) interrupt the reflective band called EZ, attributed to the outer third of the IS ellipsoid in spectral domain OCT (46, 51). The same OCT device can be used to image a preserved donor eye (**Figure 1D**). *Ex vivo* OCT, as used in this study to identify donor eyes with SDD, does reveal SDD between an undulating thin reflective line (presumed EZ) and the highly reflective RPE-basal-lamina-Bruch’s membrane band.

**Figure 2:**
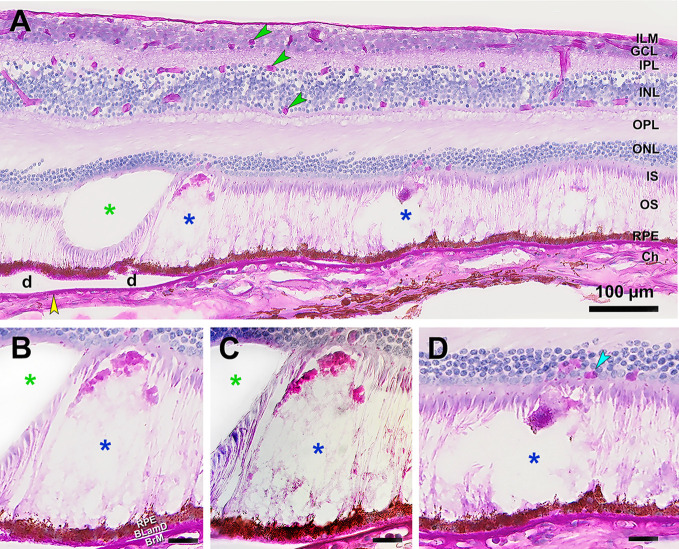
Subretinal drusenoid deposits (SDD) lack common AMD carbohydrate markers. Periodic acid Schiff hematoxylin (PASH) stained cryosection of human retina with AMD. **(A)** PASH shows retinal structure layer by layer with strong signal in retinal capillaries (green arrowheads), basal laminar deposit (BLamD), Bruch’s membrane (yellow arrowhead), and drusen (d). SDD are barely stained (blue asterisk) and are recognized primarily by the absence of photoreceptors in a dome- or flame-shaped space. The green asterisk indicates an artifactual empty space. **(B)** BLamD and Bruch’s membrane stain strongly, as does a cap of presumed outer segment fragments in stage 3 SDD (8). **(C)** Differential contrast microscopy shows a filmy material in SDD whereas an artifactual layer separation clearly lacks contents (green asterisk). **(D)** Stage 2 SDD shows one cell with RPE granules at the SDD apex. There are also other unknown PAS+ cells (cyan arrowhead) in the overlying ONL. Scale bars BCD, 20 µm; BrM, Bruch’s membrane; Ch, choroid. GCL, Ganglion cell layer; ILM, internal limiting membrane; INL, inner nuclear layer; IPL, inner plexiform layer; IS, inner segments of photoreceptors; ONL, outer nuclear layer; OPL, outer plexiform layer; OS, outer segments of photoreceptors; RPE, retinal pigment epithelium.

**Figure 2** shows how SDD are recognized in cryosections of eyes prepared for IMS. Periodic acid Schiff (PAS) histochemistry reveals strong signal in drusen, BLamD, Bruch’s membrane, and retinal vessels, with no signal in most SDD. In sections of attached retina, SDD can be recognized by an unstained dome- or flame-shaped space among the photoreceptor outer segments. Differential interference contrast microscopy reveals within these spaces a filmy content that accounts for lipid signals detected by MALDI IMS (see below). Larger deposits (stage 3, **Figures 2B, C**) may have a PAS-positive cap, attributable to outer segment fragments by the presence of disks (8). In representative cryosections of donor eyes from the same tissue source, 54%, 23% and 23% of SDD (n=199 total) were clinical stages 1, 2, and 3, respectively.

### Transmission electron microscopy of SDD and AMD deposits

**Figure 3:**
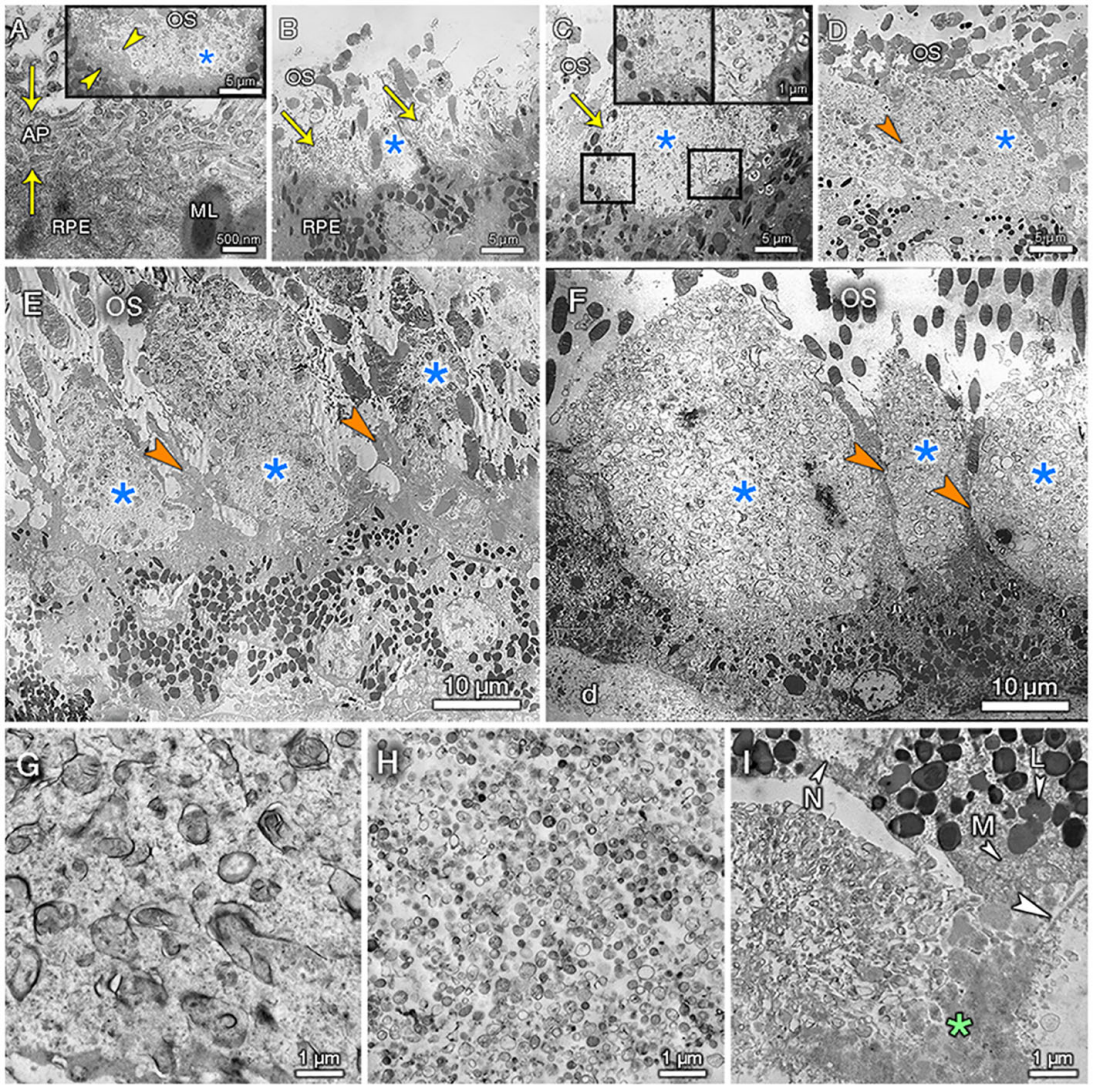
Ultrastructural stages of subretinal drusenoid deposits. **(A–I)** are from Cases 1, 2, and 3, respectively. Retina was detached from RPE and SDD in **(A–D)**. Photoreceptor outer segments (OS) are cut in cross-section due to compaction (bending). **(A)** Inset: the smallest SDD (blue asterisk) are surrounded by compressed RPE apical processes (AP, between yellow arrowheads). Main panel, RPE AP are compressed under SDD. **(B)** A small SDD is surrounded on 3 sides by AP (arrows). **(C)** Transition showing presence (left inset) and loss (right inset) of AP. **(D)** SDD inner surface meets OS without transition. The RPE cell body lacks AP and instead exhibits upward protrusions of cytoplasm lacking melanosomes (orange arrowhead). **(E)** SDD are contacted by these protrusions (orange arrowheads). OS contact the cell body and protrusions. Vertical lines surrounding OS (compare to **(A–D, F)** which lack them) are wrinkles in epoxy resin. **(F)** SDD are separated by protrusions (arrowheads) that maintain contact with OS containing visible disks. RPE is thinned with reduced packing density of electron-dense lipofuscin/melanolipofuscin granules. d, soft druse. **(G)** SDD shows irregularly shaped whorls with electron-dense membrane-like exteriors, some with partial contents, in a flocculent background. **(H)** Soft drusen have numerous lipoprotein particles with moderately electron dense interiors and non-descript background material. I, basal mounds are soft drusen material trapped within basal laminar deposits (asterisk). Arrowhead, native basal lamina of RPE. M, mitochondria; L, lipofuscin; N, nucleus.

Since the interpretation of lipid signals detected by MALDI IMS would benefit from new information about lipid-containing structures in SDD in relation to membranes of surrounding cells, we examined SDD by transmission electron microscopy. **Figures 3B–D** shows SDD at clinical stage 1 and a pre-clinical stage 0 of smaller deposits (A). In these examples SDD did not contact photoreceptor inner segments (EZ of clinical OCT). These figures support previous observations that SDD begin at the RPE and build toward photoreceptors. We extend these findings by linking SDD initiation and growth to the demise of the RPE apical processes. In a normal eye (not shown), hundreds of delicate apical processes emanate upward from RPE cell bodies along the outer segments. In pre-clinical SDD (**Figures 3A, B**) apical processes are abundant and compressed into a mat surrounding the deposit on three sides, with the deposit internal surface still contacting outer segments. In a stage 1 SDD (**Figures 3C–F**), the gradual loss of apical processes across the SDD base is apparent. The RPE maintains a contact, albeit aberrant, with outer segments via upward prolongations of cell body cytoplasm (**Figures 3D–F**). In all examples, boundaries between SDD and outer segments above and RPE cell body below are sharp (**Figure 3F**) (10). Like others, we observed no evidence for a transition zone between surrounding structures and SDD (52).

**Figures 3G–I** compares, at higher magnification, SDD ultrastructure to that of other extracellular deposits associated with AMD progression, in the same eye. In **Figure 3G**, SDD internal structure shows polydisperse electron-dense membrane-like structures (“whorls”), many wrapped around a moderately electron-dense interior. These structures are evenly distributed across the deposit, embedded within a flocculent and presumably proteinaceous background material. These structures are consistent with the appearance in high-resolution light microscopy (8, 11, 12). Histochemistry has established that SDD contains unesterified cholesterol whereas soft drusen contain both unesterified and esterified cholesterol, a hallmark core lipid of lipoprotein particles (9, 11, 12, 53). The latter are, in turn, detected ultrastructurally in human Bruch’s membrane and by isolation and direct assay (54–57). SDD whorls (**Figure 3G**) are larger and more widely spaced (with more background material) than the numerous smaller lipoprotein particles in soft drusen (**Figure 3H**). Basal mounds (**Figure 3I**) represent soft drusen material trapped between the basal RPE plasma membrane and the native basal lamina. These resemble a compressed version of soft druse material, distinct from SDD on the other side of the RPE cell body. Thus, electron microscopy confirms that SDD lipid content is distinct from surrounding cells and from other high-risk AMD deposits.

### MALDI IMS analysis

**Figure 4:**
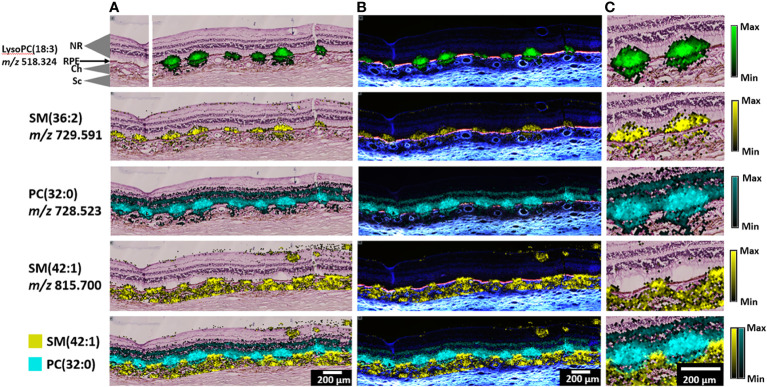
Representative IMS images, positive ion mode, from Donor 1. **(A)** Annotated H&E-stained image (top left) and lipid assignments for IMS signals (green, yellow or cyan) with overlays of lipid IMS images on top of an H&E image. **(B)** Overlay of lipid IMS images on top of an autofluorescence image. **(C)** Zoomed-in area of overlaid H&E and MALDI IMS images. Scale bars, 200 µm. Color scales indicate maximum intensity for each lipid.

To characterize deposit lipid composition, MALDI IMS and nLC-MS/MS analyses were applied to SDD in two eyes of two different donors (Donor 1, 91-year-old male; Donor 2, 87-year-old female). MALDI IMS provides semi-quantitative information on individual lipids across tissue sections whereas nLC-MS/MS provides structural information used to assign lipid identities. First, we manually inspected the IMS images for SDD-related spatial distributions. Subsequently, we performed a more exhaustive, automated computational search for SDD-related marker candidates across the 1873 detected IMS features (see below). **Figure 4** shows representative positive ion images from lipid classes detected in SDD of Donor 1. Ion images of lipids from the same class with fatty acid chains of different lengths, as well as supporting data from Donor 2 (biologic replicate) are shown in **Figures S2–6**. Lipid identifications based on nLC-MS/MS data can be found in **Tables S2** and **S3**. Overlays of MALDI IMS images with images of H&E-stained tissue (**Figure 4A**) and AF images (**Figure 4B**) clearly demonstrate the presence of SDD between the RPE and photoreceptors. LysoPC(18:3) and SM(36:2) are detected within SDD and in the inner choroid below these deposits, possibly in the choriocapillaris microvasculature. In contrast, PC(32:0) appears in SDD and in other retinal layers, most notably in photoreceptor inner and outer segments (known as the bacillary layer). SM(42:1) appears in RPE cells between individual SDD suggesting that RPE underlying SDD differ in composition (**Figure 4C**) from adjacent non-involved RPE.

LysoPCs are produced by the phospholipase A2 family of enzymes that remove a fatty acid from phosphatidylcholine. Multiple lysoPC species are detected in SDD with very little signal elsewhere except for inner choroid below SDD (**Figure 4**, top row; **Figure S2**). Similar signals were observed in SDD from Donor 2 (**Figure S3**) but not in the choroid, possibly due to lower overall signal in this tissue. LysoPC signals were also observed in SDD next to the optic nerve head (peripapillary region) (11, 58), an area where SDD are commonly observed (58, 59).

While lysoPC species were highly localized to SDD, intact (enzymatically unprocessed) PC lipids were detected in other retinal layers, as well as in SDD. PC (32:3) (**Figures S4, S5**) and PC (32:0) (**Figure 4**) show abundant signals in SDD and the bacillary layer. Other intact PC lipids display similar localizations in Donor 1 and Donor 2 (**Figures S4, S5**). **Figure S5** also shows that these lipids occur in peripapillary SDD.

**Figure 4** also displays the markedly disparate localizations of two sphingomyelin lipids. The SM (36:2) signal was strong in SDD and underlying choroid. The SM (42:1) signal, while absent from SDD, was detected in the bacillary layer between SDD and throughout the choroid, possibly involving both choriocapillaris microvasculature below Bruch’s membrane and a macro-vascular ecosystem, above the sclera. These distinct patterns are clear in an overlay of SM (42:1) and PC (32:0) (**Figure 4**, bottom row). Other SM lipids are also present in SDD and the choroid (**Figure S6**). In Donor 2 (**Figure S7**), SM lipids are similarly distributed but with higher intensity in SDD than in choroid.

**Figure 5:**
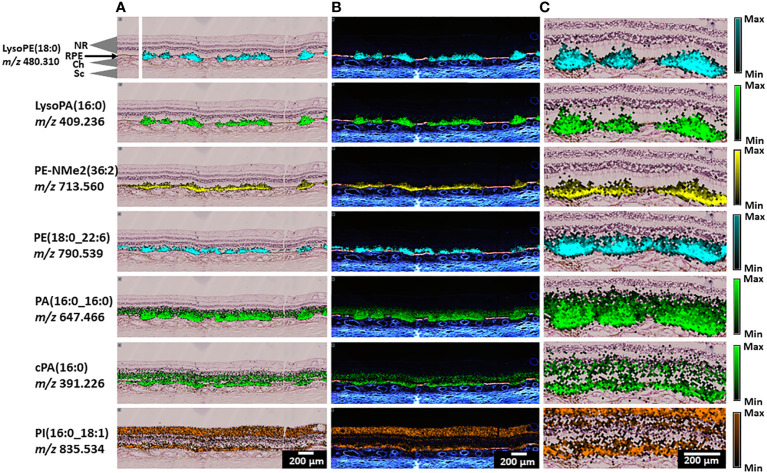
Representative IMS images, negative ion mode, from Donor 1. **(A)** Annotated H&E-stained image (top left) and lipid assignments for IMS signals with overlays of lipid IMS images on top of an H&E image. **(B)** Overlay of lipid IMS images on top of an autofluorescence image. **(C)** Zoomed-in area of overlaid H&E and MALDI IMS images. Scale bars: 200 μm. Color scale indicates maximum intensity for each lipid.

MALDI IMS analysis in negative ion mode yielded similar results to the positive ion mode analysis, with many signals abundant in SDD exclusively and others abundant in both SDD and surrounding tissue layers. **Figure 5** displays IMS data for 7 lipid classes exemplified by LysoPE(18:0), LysoPA(16:0), PE-NMe2(36:2), PE(18:0_22:6), PA(16:0_16:0), cyclic phosphatidic acid cPA(16:0), and PI(16:0_18:1). For each of these 7 classes, other species with different fatty acid chain lengths are shown in **Figures S8–17**, respectively.

**Figure 5** (top row) shows that LysoPE (18:0) is highly localized to SDD and underlying RPE. Other LysoPE species identified as (18:0), (18:1), and (20:1) were observed in SDD while LysoPE (22:6) with a docosahexaenoate side chain could be seen in inner retina as well as in SDD (**Figure S8**). In Donor 2 (**Figure S9**), these signals show similar distributions. An intact PE (18:0_22:6) present in photoreceptor outer segments (28) was observed with high abundance in SDD, RPE, and, at slightly lower intensity, in the bacillary layer **Figure 5** (fourth row). Other intact PE species show similar localization (**Figure S10**). Thus, most intact PE species are abundant in SDD and adjacent photoreceptors. In Donor 2 (**Figure S11**), signals for PE (16:0_18:2) and PE (16:0_18:1) are present in SDD and inner retina while other PE species localize in the bacillary layer and only negligibly within SDD.

Another lysolipid species in **Figure 5** (second row), LysoPA (16:0), has a distribution similar to that observed for LysoPE species with high abundance in SDD and underlying RPE. Two other LysoPAs (18:0,18:1) showed similar distributions (**Figure S12**). Results from Donor 2 were similar, however, LysoPA (18:0) also appeared throughout the inner retina (**Figure S13**). An intact PA in **Figure 5** (fifth row), PA (16:0_16:0), has strong signal in SDD and underlying RPE. Its localization also extends above the SDD into the bacillary layer. The bioactive lipid, cyclic phosphatidic acid cPA(16:0), exhibits strong signal in RPE below SDD and in the ONL, and not in SDD. However, in Donor 2 (**Figure S13**), cPA(16:0) appears in SDD and inner retina. In **Figure 5**, no negative ionization signals localize to choroid as clearly as some positive ionization signals in **Figure 4**.

PE-NMe2 (36:2) is a sphingomyelin-related species seen in negative ion mode (**Figure 5**, third row). It is formed from sphingomyelin SM (36:2), observed in positive ion mode (**Figure 4**). Both lipids show similar localization in SDD and RPE. Another PE-NMe2 lipid (16:0_16:0) had strong signal in the RPE, lesser but still substantial signal associated with SDD, and lesser but still detectable signal in the bacillary layer (**Figure S14**). In Donor 2 (**Figure S15**), PE-NMe2 signals show similar distributions with higher intensity in SDD.

Other lipids, not localizing to SDD, included some that localized to inner retina. A phosphatidylinositol (PI) species (**Figure 5**, bottom row), PI (16:0_18:1), is detected in inner retina, i.e., the NFL, in addition to the RPE layer. **Figure S16** shows that PI (16:0_20:4) is detectable in the ONL and OPL above SDD, but not in SDD. Further, PI (18:0_20:4) was observed in the ONL, INL and GCL with high abundance. Signal in other retinal layers, SDD, and RPE was lower. Similar distributions were observed with higher signal intensity in Donor 2 in **Figure S17**, with little signal in SDD.

### Automated discovery of SDD marker candidates

The preceding results were obtained by manual exploration of the massively multivariate datasets measured by IMS. However, with 983 and 890 ions detected in positive and negative ion mode, respectively, manual examination of all 1873 individual ion images to assess a relationship to SDD is impractical. Also, even if the number of measured ion images allows for manual examination, human bias and drift may still affect a manual search for SDD-markers. One could argue that the number of ion species could be reduced beforehand by using prior knowledge or by narrowing the focus to specific molecular classes. However, such a targeted approach would negate the discovery advantage of IMS to potentially reveal molecular species whose tissue distribution suggests a previously unknown relationship to SDD.

To reduce the impact of these issues, while still permitting an exhaustive search for potential SDD markers, we supplemented manual search with an automated computational search based on supervised machine learning and subsequent interpretation of the learned IMS-to-SDD model. The method was developed by authors LT, LGM, and RV and demonstrated in the context of murine kidney tissue (36); however, to our knowledge, this is the first application to human eye IMS data. The goal of using an automated method for SDD marker candidate discovery is to inform on the mechanism of deposit formation.

This approach entails two steps. First, we built a multivariate classification model that takes a vector of 983 (or 890) features as input and that predicts whether an IMS pixel is a member of SDD tissue. In both IMS ionization modes, strong SDD-prediction capability was achieved with an F1-score (evaluation metric to measure model accuracy using precision and recall) of 0.9823 (positive ion mode) and 0.9854 (negative ion mode). Once we had a computational model that recognized SDD based on IMS signals, we used Shapley additive explanations (SHAP) to explore how this model made SDD predictions. SHAP analysis quantified the contribution of each input feature to the model’s output prediction (60). A local SHAP importance score measures the influence of a feature on model predictions for a particular pixel. The global SHAP importance score measures the influence of a feature on model predictions for all pixels in the dataset. Features with highest estimated relevance to recognizing SDD, i.e., whose increase (or decrease) in ion intensity helps in this recognition task, will tend to be at the top of this ranking. As such, the two-step method offers an automated means of obtaining, from hundreds of recorded ions, a shortlist of potential candidate markers for SDD, worthy of further investigation. Note that candidates discerned in this way can be either positively or negatively correlated with SDD. Furthermore, isotopes were retained in the feature list, so multiple isotopes of the same molecular species can appear in the shortlist.

**Figure 6:**
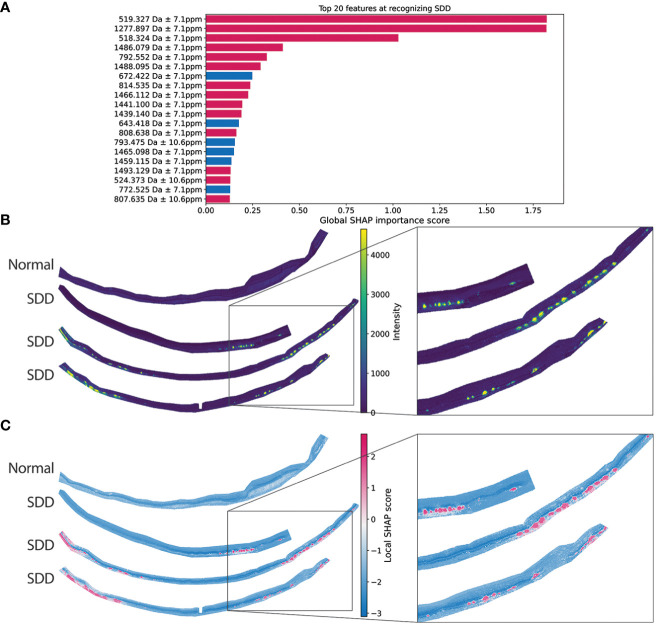
SDD marker candidate discovery in positive ion mode. **(A)** Global feature importance of the 20 m/z values most relevant to recognizing SDD, as per the SHAP interpretability method. The top two positive-mode features relevant to SDD are m/z 519.327 and m/z 1277.897. Red bars indicate that an increase in ion intensity for these ions increases the probability that there is SDD present. Blue bars indicate that these signals are relevant markers for SDD in that their ion intensity decreases in SDD tissue areas. **(B)** IMS images of the top-ranked feature (m/z 519.327) across multiple retina tissue samples (top tissue sample “normal” showed no SDD present by OCT and histology; bottom two samples are from the same donor). **(C)** SHAP maps, reporting localized SHAP importance of the top ranked feature (m/z 519.327), show a strong positive correlation between ion intensity and SDD.

**Figure 6** shows the results of applying the automated SDD marker candidate discovery method to the positive ion mode MALDI IMS data. The bar plot in **Figure 6A** shows only the top 20 of ranked 983 detected ions in terms of their global feature importance, i.e., ions most relevant to recognizing SDD as per the SHAP interpretability method. The top two features are m/z 519.327 ± 7.1 ppm, identified as the ^13^C isotope of LysoPC(18:3), and m/z 1277.897 ± 7.1 ppm, which is an unknown positively correlating candidate marker for SDD. Although not identified, the estimated potential for m/z 1277.897 to be an SDD marker becomes apparent when its ion image is perused (**Figure S18**). This signal clearly and specifically localizes to SDD tissue areas. The third highest scoring feature in the positive mode, m/z 518.324 ± 7.1 ppm, is the monoisotopic ion of the top-ranked feature, emphasizing that the same lipid species reported by both isotopes [LysoPC(18:3)] is SDD-relevant. As all three ions are colored red in **Figure 6A**, greater intensity for these ions increases the probability that SDD is present at the measurement location. Other ion species, such as m/z 672.422 ± 7.1 ppm, are colored blue in **Figure 6A** to indicate that they are relevant to SDD because their intensities decrease in SDD tissue areas (see **Figure S19**). The high global SHAP score ranking in **Figure 6A** suggests that m/z 519.327 has a spatial distribution that is relevant to SDD. The red bar for m/z 519.327 suggests its intensity positively correlates with SDD presence. Both observations are confirmed in **Figure 6B** where the ion images for m/z 519.327 are shown across the different retina tissue samples. The ion images show that m/z 519.327 localizes strongly to SDD. Note that no signal for m/z 519.327 was observed in the top tissue section from a retina lacking SDD as determined by OCT and histological analysis. In **Figure 6C**, a SHAP map (36) is shown for each of the m/z 519.327 ion images in **Figure 6B**. A SHAP map reports a feature’s local SHAP importance scores across all pixels, enabling a spatially localized understanding of the classifier’s decision-making process. Briefly, in the red areas of the SHAP map, the ion intensity of m/z 519.327 (regardless of whether it is low or high) increases the likelihood of SDD being present, in the blue areas its intensity reduces the likelihood of SDD presence, and in the white tissue areas m/z 519.327 seems to have no relevance to SDD recognition. Since m/z 519.327 is a positive marker candidate for SDD and nearly exclusive to SDD tissue (**Figures 5B, C**), there is a strong correlation between the ion image (showing ion intensity) and the SHAP map (showing SDD-recognition relevance). However, more complex SHAP maps can become apparent, e.g., for negatively correlating markers (e.g., see **Figures S19–21**) and for positively correlating markers that are not exclusive to SDD. Such specific SHAP map interpretations and their use cases are discussed further in Tideman et al. (36).

**Figure 7:**
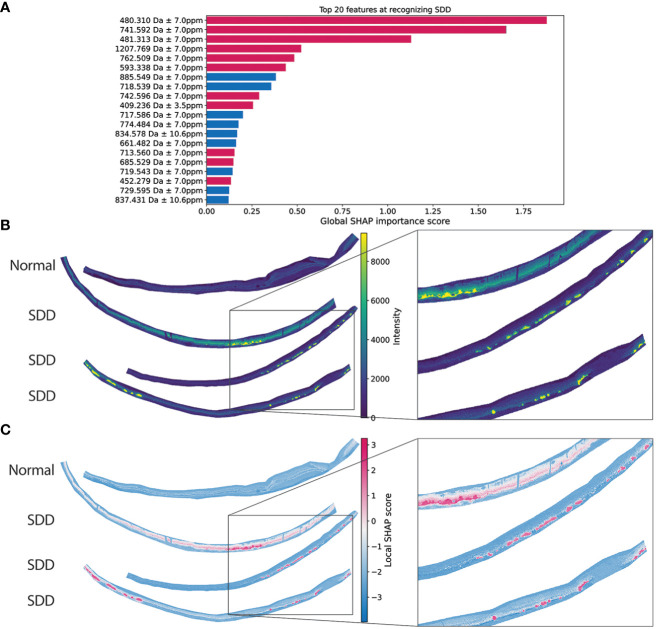
SDD marker candidate discovery in negative ion mode. **(A)** Global feature importance of the 20 m/z values most relevant to recognizing SDD, as per the SHAP interpretability method. The top two negative-mode features in terms of relevance to SDD are m/z 480.310 and m/z 741.592. Red bars indicate that an increase in ion intensity for these ions increases the probability that there is SDD present. Blue bars indicate that these signals are relevant markers for SDD in that their ion intensity decreases in SDD tissue areas. **(B)** IMS images of the top-ranked feature (m/z 480.310) across multiple retina tissue samples (top tissue sample “normal” showed no SDD present by OCT and histology; bottom two samples are from the same donor). **(C)** SHAP maps, reporting localized SHAP importance of the top-ranked feature (m/z 480.310), shows a strong positive correlation between ion intensity and SDD.

**Figure 7** shows SDD marker candidates discovered in negative ion mode. **Figure 7A** shows only the top 20 of ranked 890 detected ions of relevance to SDD, per the SHAP interpretability method. The top two features, suggesting a strong correlation (both positively correlated) with SDD, are m/z 480.310 ± 7.0 ppm, identified as LysoPE(18:0), and m/z 741.592 ± 7.0 ppm, which has yet to be identified. While not identified, the potential for m/z 741.592 as an SDD marker is apparent when its ion image is viewed (**Figure S20**). Signal for m/z 741.592 ± 7.0 ppm clearly and specifically localizes to SDD areas. The third highest scoring feature, m/z 481.313 ± 7.0 ppm, is the ^13^C isotope of the top-ranked feature. This confirms that the same lipid species reported by both isotopes [LysoPE (18:0)] is SDD-relevant. All three ions are colored red in **Figure 7A**, indicating that greater ion intensity for these ions increases the probability that SDD is present. As before, ions such as m/z 885.549 ± 7.0 ppm, are colored blue in **Figure 7A** to indicate that they are relevant markers for SDD, because their intensities decrease in SDD tissue areas (see **Figure S21**). Its high global SHAP score ranking in **Figure 7A** suggests m/z 480.310 is a positively correlating SDD-marker candidate, **Figure 7B** confirms this by showing the distribution of m/z 480.310 across multiple retina tissue samples. As suggested by the SHAP ranking, m/z 480.310 is strongly localized to SDD. Additionally, **Figure 7C** shows the SHAP map of the top-ranked m/z 480.310 ± 7.0 ppm). Here, the ion image of m/z 480.310 and its SHAP map are strongly correlated since this ion is a positive marker candidate and the measured tissue areas consist of only retina tissue in which m/z 480.310 seems to localize exclusively to SDD. Also, note that the top tissue section in **Figure 7B** (from a normal retina) lacks signal for m/z 480.310, further confirming its marker potential for SDD.

### Immunohistochemistry of phospholipase A2G5

We next investigated a potential mechanism for the production of lysoPCs. These lipids are formed when phospholipase A2 (PLA2) enzymes hydrolyze PCs and remove one acyl chain as a fatty acid. The mammalian genome encodes over 50 PLA2s and related enzymes, which are classified in four groups by structure, enzymology, and evolutionary relationships (secreted, cytosolic, Ca2+-independent, and platelet-activating factor acetylhydrolases) (61). Of these, those of possible relevance to extracellular SDD are the catalytically active isoforms of secreted PLA2 (sPLA2, ten are known). Benign fleck retina (MIM 228980) is a rare autosomal recessive condition caused by mutations in the *PLA2G5* gene (secreted phospholipase A2 group 5) (62–64). Benign fleck retinas lack visual or electrophysiologic deficits yet feature a striking pattern of diffuse, yellow-white, fleck-like fundus lesions, extending to the far retinal periphery and sparing the central macula. When viewed by spectral domain OCT, the flecks are seen as SDD identical to AMD SDD. Thus we sought to confirm expression of PLA2G5 gene product in human retina using immunohistochemistry.

**Figure 8:**
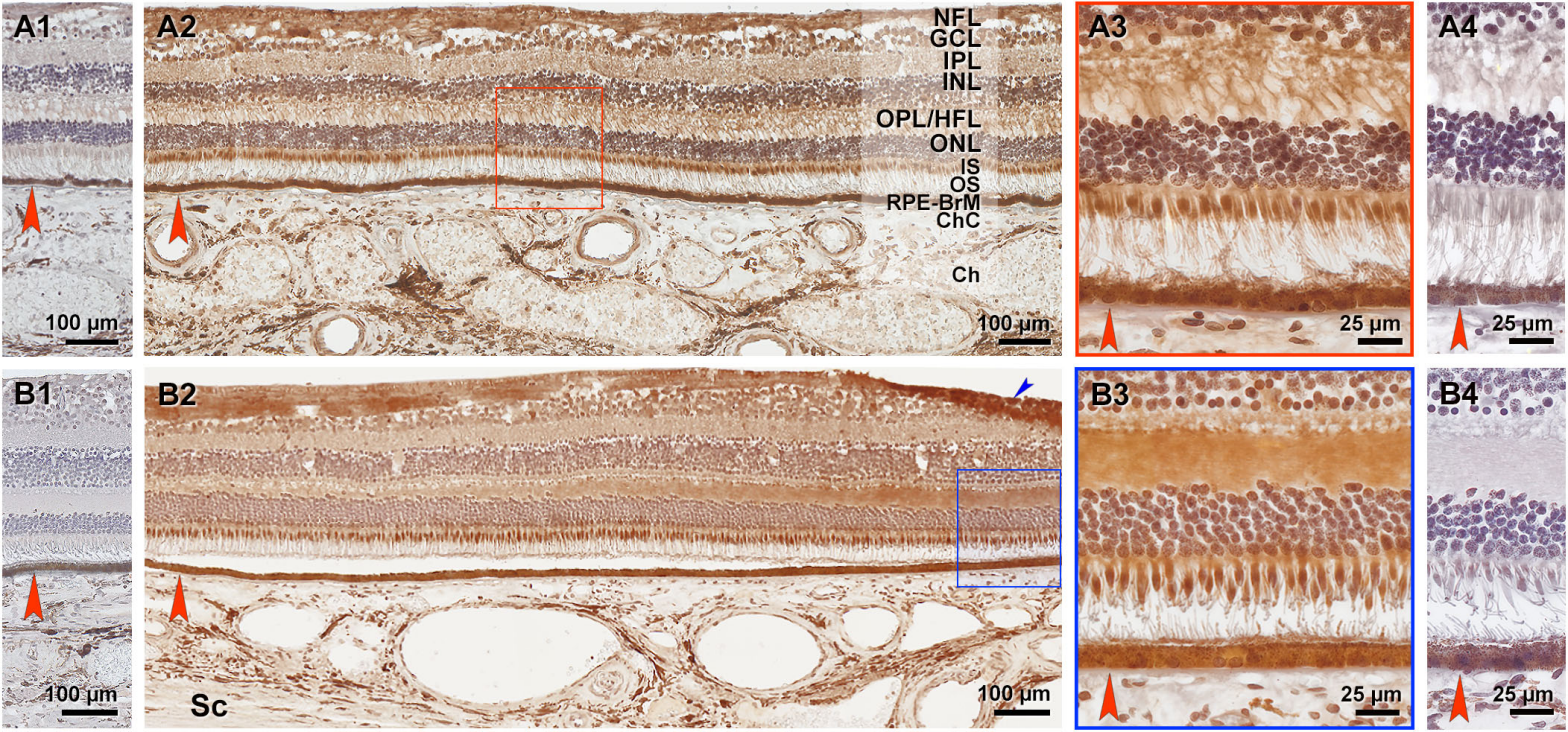
Phospholipase A2 group V (PLA2G5) immunoreactivity in human retina. Two healthy aged retinas were cryosectioned **(A1–4, B1–4)**. Sections through central retina (identified by the HFL) were immuno-stained with PLA2G5 polyclonal antibody. Reaction product is reddish brown. **(A2, B2)** and **(A4, B4)** are sham controls, neighboring sections processed without primary antibody. BrM is indicated by red arrowhead. **(A2, A3, B2, B3)** Strong PLA2G5 signal is observed in NFL, ellipsoid of photoreceptors (cones more than rods), and RPE cell bodies and apical processes. Moderate signals were observed in GCL, IPL, and cone cell bodies along the outer edge of the ONL. In **(B2)** the HFL was stained more heavily towards the fovea (at the right) than at the temporal side (at the left). Photoreceptor outer segments were unlabeled. Blue arrowhead indicates a fold. NFL, nerve fiber layer; GCL, Ganglion layer; IPL, inner plexiform layer; INL, inner nuclear layer; OPL, outer plexiform layer; HFL, Henle’s fiber layer; ONL, outer nuclear layer; IS, inner segments of photoreceptor; OS, outer segments of photoreceptors; RPE, retinal pigment epithelium; BrM, Bruch’s membrane; ChC, choriocapillaris; Ch, choroid; Sc, sclera.

**Figures 8** shows sections through central retina of two normal aged specimens. Strong PLA2G5 signal is observed in NFL, ellipsoid portion of photoreceptor inner segments (cones more than rods), and RPE cell bodies and apical processes (**Figures 8A2, 8A3, 8B2, 8B3**). Moderate signals were observed in GCL, IPL (**Figures 8A2, 8B2**), and cone cell bodies along the outer edge of the ONL (**Figures 8A3, 8B3**). The HFL was stained more heavily towards the fovea than at the temporal side (**Figure 8B2**). Photoreceptor outer segments were unlabeled (**Figures 8A2, 8A3, 8B2, 8B3**). Control experiments were negative (**Figures 8A1, 8A4, 8B1, 8B4**).

## Discussion

Retina deposits such as drusen and SDD are known risk factors for AMD, and their molecular composition and mechanisms of formation are still being learned. In this work we combined advanced, spatially resolved mass spectrometric approaches with a machine learning strategy to identify SDD lipid composition in donor eyes with AMD. Our data implicate lipid dysregulation leading to accumulation of lysolipids in SDD. Specifically, our IMS data reveal that lysolipids (LysoPC, LysoPE, LysoPA) appear almost exclusively in SDD (**Figures 4**, **5**; **S2, 3, 8, 9, 12, 13**) and that these lysolipids likely come from precursors found primarily in photoreceptors, which express a secretory phospholipase A2.

In addition to insights gained into SDD (discussed below), this study advanced a novel and automated approach to thoroughly mine SDD molecular composition. The combination of IMS with an automated marker candidate discovery method (36), powered by interpretable supervised machine learning, provided an open-ended, unbiased exploration of retinal tissues containing SDD. The SHAP-based discovery approach delivered a shortlist of potential markers from >1800 ion images by comparing the chemical content of SDD to regions of uninvolved retina. This strategy yielded SDD marker candidates including LysoPE(18:0) and LysoPC(18:3) as well as two unidentified marker candidates, namely m/z 741.592 and m/z 1277.897. These examples demonstrate that automation in this manner can be utilized not only for confirmation and exhaustive assessment, but also for generation of new hypotheses and guidance for targeted downstream analysis.

Our PLA2G5 immunolocalization study using a polyclonal antibody supports other findings with this antibody (shown at the vendor website). It also supersedes suggestions based on a monoclonal antibody that *PLA2G5* gene product localizes primarily to the OPL (62), far from SDD. Publicly available gene expression data link *PLA2G5* to photoreceptors and RPE (65, 66). Secreted phospholipase A2 enzymes require high calcium concentrations in the extracellular compartment to hydrolyze phospholipids like those in plasma membranes of activated, damaged, or dying cells, plus extracellular vesicles, lipoproteins, surfactants, and other materials. The released fatty acids are precursors to both pro-inflammatory (arachidonate, 20:4) and anti-inflammatory (eicosapentaenoate, 20:5(n-3), docosahexaenoate 22:6(n-3)) lipid mediators that either exacerbate or resolve pathophysiology, in a tissue- and context-specific manner.

Our discovery of abundant lysolipids in SDD and PLA2G5 expression in photoreceptors and RPE may direct future efforts to decipher clinically relevant lipid homeostasis in retina. Strong label in cone inner segments and in the HFL close to the fovea, in the absence of signal in Müller glia, suggests that cones might be stronger expressers than rods. While it is tempting to speculate that phospholipase PLA2G5 is important in outer retinal lipid homeostasis and its dysfunction leads to SDD formation, three points require further investigation. First, how does a mutation lead to deposits if functional PLA2G5 is required to generate lysolipids? Second, if cone expression of PLA2G5 is strong, why do SDD form where rods are numerous? Third, how are the autofluorescent flecks of PLA2G5 retinopathy reconciled with the infrequent autofluorescence of AMD SDD? (67, 68). Extending a published lipid transport barrier model for soft drusen formation (5) we suggest that remodeling of plasma membrane lipids by PLA2G5 is a constitutive outer retinal activity and that a barrier to effective removal of lysolipids is failing RPE. Finally, even though complement proteins in drusen synergized with genome wide association studies to implicate complement pathways (69, 70), AMD-like extracellular deposits were also found in monogenic inherited retinopathies (65, 71–73), thus facilitating mechanistic studies and model system building. We expect that our findings will enable similar studies for SDD-driven AMD.

The biochemical and physiological roles of lysophospholipids, including their role in human retinal health and disease, are still emerging. Markers of LysoPC metabolism are abundant in plasma of patients with neovascular AMD, suggesting involvement in AMD progression (66, 74–77). Retinal entry of LysoPCs containing docosahexaenoate, a fatty acid abundant in neural membranes and essential for vision (78), is mediated by the facilitator superfamily domain-containing protein 2 (Mfsd2a). Photoreceptors in Mfsd2a-deficient mice progressively degenerate (78). Mfsd2a also transports LysoPEs resulting from the hydrolysis of PE lipids (79); a role of LysoPEs in photoreceptor degeneration has not yet been shown (78, 80).

LysoPA lipids were observed within SDD and surrounding retina, and at lower intensities for some species, in inner retina. LysoPA lipids are generated either directly by hydrolysis of phosphatidic acid (PA) or by cleavage of lysophospholipid headgroups by autotaxin (ATX, gene *ENPP2*). This secreted protein with lysophospholipase D activity is highly expressed in human RPE (81). LysoPA can activate expression of angiogenic factors in non-ocular cells (82). When applied at high concentrations, LysoPA reduces phagocytosis of outer segments by induced pluripotent stem cell derived RPE (66).

Of sphingomyelin lipids observed, SM (42:1) strikingly avoids SDD, localizing instead to intervening RPE, while SM (36:2) localizes to deposits precisely. These findings notably exclude a straightforward conversion of outer segments to SDD. Sphingomyelin lipids are a diverse group of bioactive signaling molecules implicated in retinal degeneration (83–85), possibly indicating alteration of endosomal and autophagosomal processing by lysosomes as in Niemann Pick disease (86).

In summary, using advanced analytical methodology and computational methods, supplemented with high-quality microscopy, we demonstrated that lysolipids (LysoPCs, LysoPEs, and LysoPAs) accumulate in SDD and that their precursors (PCs, PEs, and PAs) also appear in both SDD and adjacent photoreceptors. The notion that SDD content derives primarily from photoreceptors is consistent with cone opsin immunoreactivity in SDD (12). Our ultrastructural observations indicate that SDD do not contain recognizable outer segment disks but rather whorls, suggestive of substantial modification. The formation of lysolipids in SDD could occur either through phospholipase activity, including PLA2G5, or through non-enzymatic hydrolysis over time (87). The sharp boundaries between SDD and surrounding OS and RPE apical processes seen by electron microscopy suggest that this hydrolysis occurs in the extracellular space.

Key questions for future research are the functional consequences of lysolipid accumulation. Given that lysolipids participate in signaling, neural development, and inflammation (61, 80), they likely play multiple roles in retinal health and AMD progression. Understanding these roles could lead to targets for new therapies and preventions.

## Data availability statement

The original contributions presented in the study are included in the article/**Supplementary Material**, further inquiries can be directed to the corresponding author.

## Ethics statement

The studies involving humans were approved by Institutional Review Board of the University of Alabama at Birmingham. The studies were conducted in accordance with the local legislation and institutional requirements. The human samples used in this study were acquired from Advancing Sight Network eye bank. Written informed consent for participation was not required from the participants or the participants’ legal guardians/next of kin in accordance with the national legislation and institutional requirements.

## Author contributions

KS: Conceptualization, Funding acquisition, Project administration, Supervision, Writing – original draft, Writing – review & editing. DA: Data curation, Formal analysis, Investigation, Methodology, Writing – original draft, Writing – review & editing. AK: Data curation, Formal analysis, Investigation, Methodology, Writing – original draft, Writing – review & editing. LM: Formal analysis, Investigation, Methodology, Software, Writing – original draft, Writing – review & editing. NP: Investigation, Methodology, Software, Writing – review & editing. LT: Methodology, Software, Writing – review & editing. DC: Data curation, Formal analysis, Investigation, Writing – original draft, Writing – review & editing. BA: Data curation, Investigation, Methodology, Writing – review & editing. JM: Data curation, Formal analysis, Investigation, Methodology, Writing – review & editing. TA: Funding acquisition, Writing – review & editing. ST: Formal analysis, Investigation, Software, Writing – review & editing. RV: Conceptualization, Project administration, Supervision, Writing – original draft, Writing – review & editing. CC: Conceptualization, Formal analysis, Funding acquisition, Project administration, Supervision, Writing – original draft, Writing – review & editing.

## Glossary

**Table:**

AMD	age related macular degeneration
AP	apical processes
ATX	autotaxin
BLamD	basal laminar deposit
BrM	Bruch’s membrane
BuOH	butyl alcohol
BEH	Ethylene Bridged Hybrid
Ch	choroid
CMC	carboxymethylcellulose
cPA	cyclic phosphatidic acid
d	drusen
DAN	1,5-diaminonaphthalene
DHA	2,6-dihydroxyacetophenone
DIC	differential interference contrast
ELM	external limiting membrane
ESI	electrospray ionization
EZ	ellipsoid zone
FA	fatty acid
GCL	Ganglion cell layer
GL	glycerolipids
GP	glycerophospholipids
HPLC	high performance liquid chromatography
ICL	inner collagenous layer
ILM	internal limiting membrane
IPA	isopropyl alcohol
IMS	imaging mass spectrometry
INL	inner nuclear layer
IPL	inner plexiform layer
IS	inner segments of photoreceptors
ITO	indium tin oxide
L	lipofuscin
LysoPA	lysophosphatidic acid
LysoPC	lysophosphatidylcholine
LysoPE	phosphatidylethanolamine
M	mitochondria
MALDI IMS	Matrix-assisted laser desorption ionization imaging mass spectrometry, ML, melanolipofuscin
MMC	mixture of methanol/chloroform
MS	mass spectrometry
MTEB	methyl tert-butyl ether
MUFA	mono-unsaturated fatty acid
nLC-MS/MS	nano liquid chromatography tandem mass spectrometry
NCE	normalized collision energy
N	nucleus
NFL	never fiber layer
OCT	optical coherence tomography
ONL	outer nuclear layer
OPL	outer plexiform layer
OS	outer segments of photoreceptors
OTAP	osmium tannic acid paraphenylenediamine
Ox-LDL	oxidized low-density lipoprotein
PAS	periodic acid Schiff
PASH	periodic acid Schiff hematoxylin
PC	phosphatidylcholine
PE-NMe2	1,2-Dipalmitoyl-sn-glycero-3-phospho-N,N-dimethylethanolamine
PE	phosphatidylethanolamine*;* PFA, paraformaldehyde
PI	phosphatidyl inositol
PLA1	phospholipase A1
PLA2	phospholipase A2
R	retina
ROI	regions of interest
ROS	reactive oxygen species
RPE	retinal pigment epithelium
RPE-BL-BrM	RPE-basal lamina-Bruch’s membrane band
RPE-BL	RPE basal lamina
QTOF	quadrupole time-of-flight mass spectrometer
Sc	sclera
SDD	subretinal drusenoid deposits, also called reticular pseudodrusen (RPD)
SHAP	Shapley Additive exPlanations
SM	sphingomyelin
TEM	transmission electron microscopy
TIC	total ion current
TOF	time-of-flight mass spectrometer
