# Supplementary material for: Lysolipids are prominent in subretinal drusenoid deposits, a high-risk phenotype in age-related macular degeneration
Source: Front Ophthalmol (Lausanne). 2023 Nov 24;3:1258734. doi: 10.3389/fopht.2023.1258734 (PMC10769005; doi:10.3389/fopht.2023.1258734)
Supplement: Supplementary file 1 [file DataSheet_1.pdf]

Lysolipids are prominent in subretinal drusenoid deposits, a high-risk phenotype in age-related macular degeneration

David M.G. Anderson*[1], Ankita Kotnala*[1,2], Lukasz G. Migas[3], N. Heath Patterson[1], Léonore E.M. Tideman[3], Dongfeng Cao[2], Bibek Adhikari[4], Jeffrey D. Messinger[2], Thomas Ach[5], Sara Tortorella[6], Raf Van de Plas[1,3], Christine A. Curcio[2] and Kevin L. Schey[1]

1. Department of Biochemistry, Vanderbilt University, Nashville TN
2. Department of Ophthalmology and Visual Sciences, University of Alabama at Birmingham, Birmingham AL, USA
3. Delft Center for Systems and Control (DCSC), Delft University of Technology, Delft, Netherlands
4. Vision Science Graduate Program, University of Alabama at Birmingham, Birmingham AL, USA
5. Department of Ophthalmology, University Hospital Bonn, Bonn, Germany
6. Molecular Horizon Srl, Via Montelino 30, 06084 Bettona, Perugia, Italy

Supplementary Abbreviation, Figures, Tables, Materials and Methods

Abbreviations 2
Figure S1. Workflow of sample preparation and multimodal imaging........ 3
Figure S2. IMS of LysoPC lipids, Donor 1, positive ion mode. 5
Figure S3. IMS of LysoPC lipids, Donor 2, positive ion mode. 6
Figure S4. IMS of PC lipids, Donor 1, positive ion mode. 7
Figure S5. IMS of PC lipids, Donor 2, positive ion mode. 8
Figure S6. IMS of SM and PC lipids, Donor 1, positive ion mode. 9
Figure S7. IMS of SM and PC lipids, Donor 2, positive ion mode. 10
Figure S8. IMS of LysoPE lipids, Donor 1, negative ion mode. 11
Figure S9. IMS of LysoPE lipids, Donor 2, negative ion mode. 12
Figure S10. IMS of PE lipids, Donor 1, negative ion mode. 13
Figure S11. IMS of PE lipids, Donor 2, negative ion mode. 14
Figure S12. IMS of LysoPA and PA lipids, Donor 1, negative ion mode. 15
Figure S13. IMS of LysoPA and PA lipids, Donor 2, negative ion mode. 16
Figure S14. IMS of PE-NMe2 lipids, Donor 1, negative ion mode. 17
Figure S15. IMS of PE-NMe2 lipids, Donor 2, negative ion mode. 18
Figure S16. IMS of PI lipids, Donor 1, negative ion mode. 19
Figure S17. IMS of PI lipids, Donor 2, negative ion mode. 20
Figure S18. IMS images and SHAP maps of m/z 1277.897. 21
Figure S19. IMS images and SHAP maps of m/z 672.422. 22
Figure S20. IMS images and SHAP maps of m/z 741.592. 23
Figure S21. IMS images and SHAP maps of m/z 885.549. 24
Table S1: Donor eyes used in figures 25
Table S2. Tandem mass spectral fragmentation patterns, positive ion mode. 26
Table S3 Tandem mass spectral fragmentation pattern, negative ion mode........ 27
Supplemental Materials and Methods 30
Supplemental Information References 40

Abbreviations

AMD, age related macular degeneration; AP, apical processes; ATX, autotaxin; BLamD, basal laminar deposit; BrM, Bruch's membrane; BuOH, butyl alcohol; BEH, Ethylene Bridged Hybrid; Ch, choroid; CMC, carboxymethylcellulose; cPA, cyclic phosphatidic acid; d, drusen; DAN, 1,5-diaminonaphthalene; DHA, 2,6-dihydroxyacetophenone; DIC, differential interference contrast; ELM, external limiting membrane; ESI, electrospray ionization; EZ, ellipsoid zone; FA, fatty acid; GCL, Ganglion cell layer; GL, glycerolipids; GP, glycerophospholipids; HPLC, high performance liquid chromatography; ICL, inner collagenous layer; ILM, internal limiting membrane; IPA, isopropyl alcohol; IMS, imaging mass spectrometry; INL, inner nuclear layer; IPL, inner plexiform layer; IS, inner segments of photoreceptors; ITO, indium tin oxide; L, lipofuscin; LysoPA, lysophosphatidic acid; LysoPC, lysophosphatidylcholine; LysoPE, phosphatidylethanolamine; M, mitochondria; MALDI IMS, Matrix-assisted laser desorption ionization imaging mass spectrometry, ML, melanolipofuscin; MMC, mixture of methanol/chloroform; MS, mass spectrometry; MTEB, methyl tert-butyl ether; MUFA, mono-unsaturated fatty acid; nLC-MS/MS, nano liquid chromatography tandem mass spectrometry; NCE, normalized collision energy; N, nucleus; NFL, never fiber layer; OCT, optical coherence tomography; ONL, outer nuclear layer; OPL, outer plexiform layer; OS, outer segments of photoreceptors; OTAP, osmium tannic acid paraphenylenediamine; Ox-LDL, oxidized low-density lipoprotein; PAS, periodic acid Schiff; PASH, periodic acid Schiff hematoxylin; PC, *phosphatidylcholine;* PE-NMe2, 1,2-Dipalmitoyl-sn-glycero-3-phospho-N,N-dimethylethanolamine; PE, *phosphatidylethanolamine;* PFA, paraformaldehyde; PI, phosphatidyl inositol; PLA1, phospholipase A1; PLA2, phospholipase A2; R, retina; ROI, regions of interest; ROS, reactive oxygen species; RPE, retinal pigment epithelium; RPE-BL-BrM, RPE-basal lamina-Bruch's membrane band; RPE-BL, RPE basal lamina; QTOF, quadrupole time-of-flight mass spectrometer; Sc, sclera; SDD, subretinal drusenoid deposits, also called reticular pseudodrusen (RPD); SHAP, Shapley Additive exPlanations; SM, sphingomyelin; TEM, transmission electron microscopy; TIC, total ion current; TOF, time-of-flight mass spectrometer.

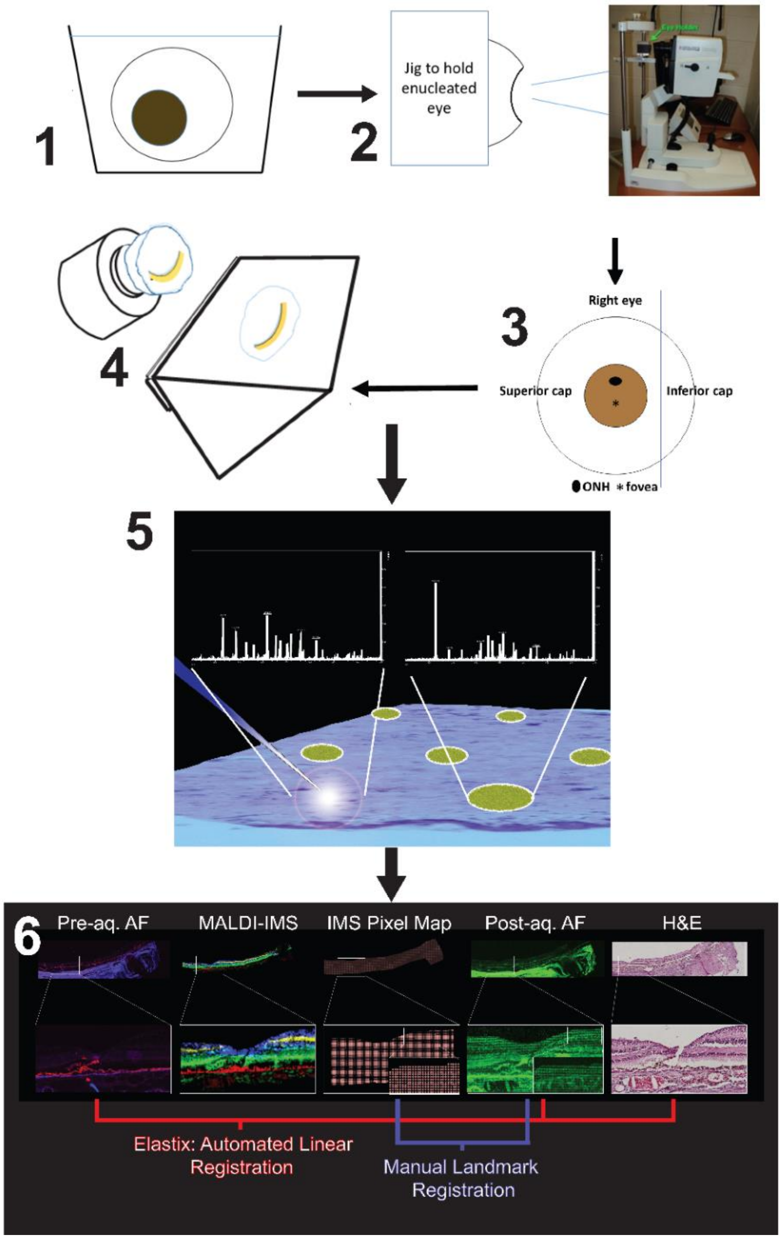

Figure S1. Workflow of sample preparation and multimodal imaging.
1. Whole eye mounted in jig to obtain OCT image. **2.** The cornea is removed before fixation with 4% paraformaldehyde (PFA) overnight and 1% PFA for 48 hours at 4℃. **3.** The eye is place in a dissection guide as described in the text to capture a belt with the optic nerve, macula, and temporal periphery. The belt is embedded in 2.25% CMC in a cryomold. The caps are discarded. **4.** Cryosections at 12-14 µm throughout the entire eye are thaw-mounted on either glass or ITO slides. **5.** ITO slides are imaged for autofluorescence (AF) before being coated with matrix via sublimation for acquisition of IMS data. **6.** Highly accurate data registration is performed from IMS, post-acquisition AF, pre AF, and H&E stained tissue.

Reprinted with permissions from “Anderson DMG, Messinger JD, Patterson NH, Rivera ES, Kotnala A, Spraggins JM, et al. Lipid Landscape of the Human Retina and Supporting Tissues Revealed by High-Resolution Imaging Mass Spectrometry. J Am Soc Mass Spectrom. 2020;31(12):2426-36.”Copyright 2020 American Chemical Society.

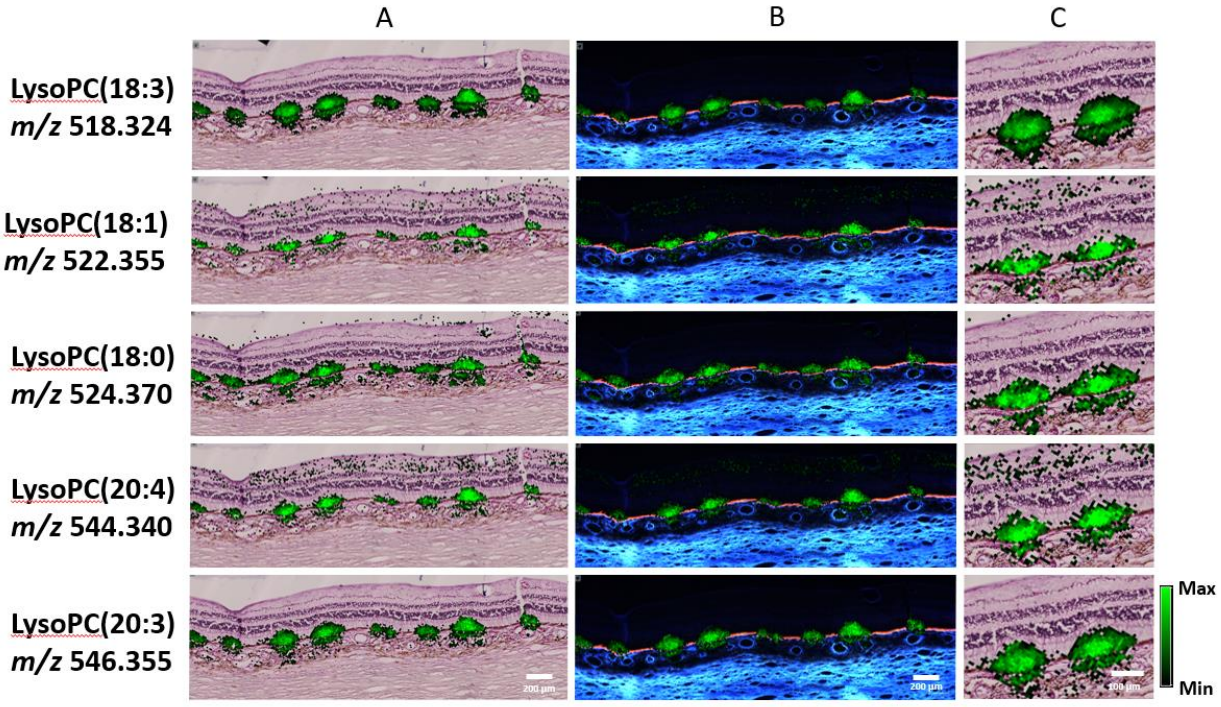

Figure S2. IMS of LysoPC lipids, Donor 1, positive ion mode.
Overlays of IMS images with H&E (A, C) and AF (B) images show that signals for LysoPC lipids (green) are concentrated in SDD. Scale bars apply to entire column: A, B, 200 µm; C, 100 µm. See also Figure 4.

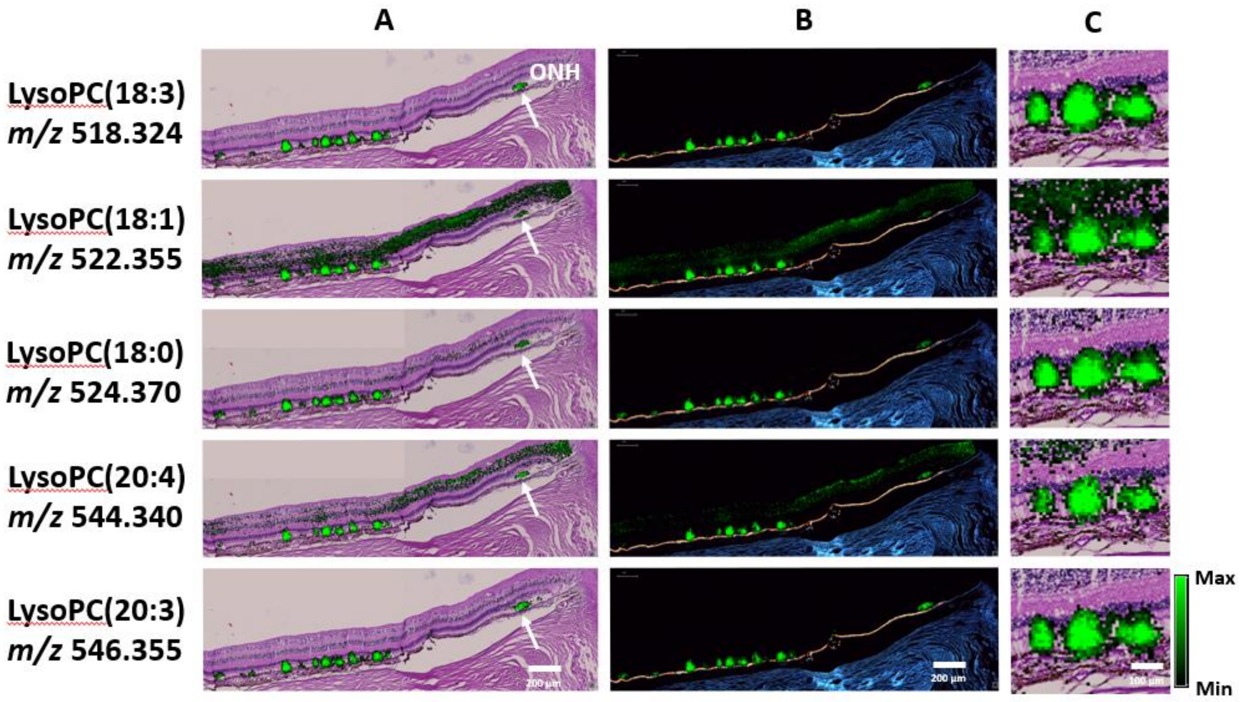

Figure S3. IMS of LysoPC lipids, Donor 2, positive ion mode.
Overlays of IMS images with H&E (A, C) and AF (B) images show that signals for LysoPC lipids (green) are concentrated in SDD. White arrow indicates peripapillary SDD, next to the optic nerve head. Scale bars apply to entire column: A, B, 200 µm; C, 100 µm. See also Figure 4.

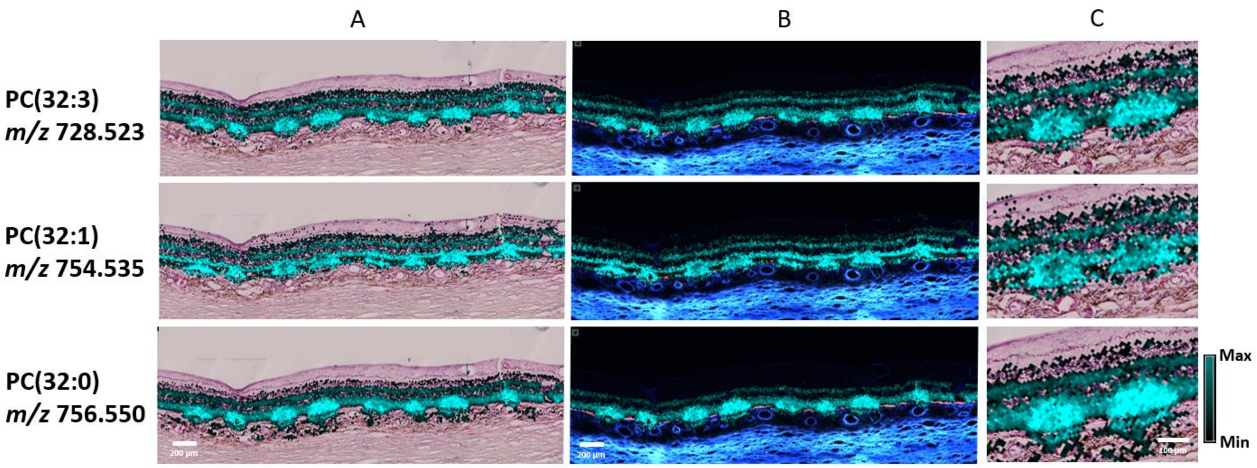

Figure S4. IMS of PC lipids, Donor 1, positive ion mode.
Overlays of IMS images with H&E (A, C) and AF (B) images show that signals for PC lipids (cyan) are more intense in SDD than elsewhere. Scale bars apply to entire column: A, B, 200 µm; C, 100 µm. See also Figure 4.

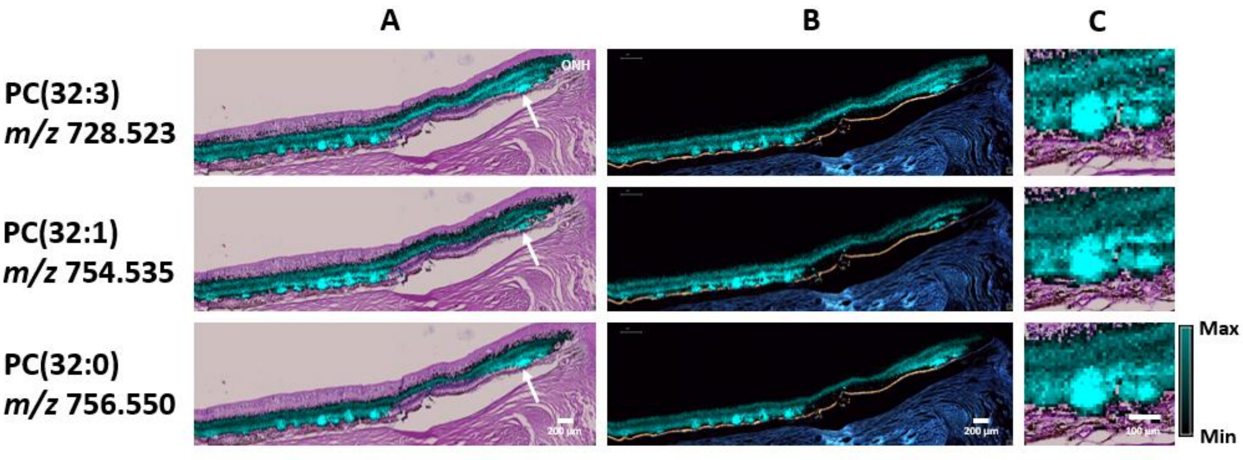

Figure S5. IMS of PC lipids, Donor 2, positive ion mode.
Overlays of IMS images with H&E (A,C) and AF (B) images show that signals for PC lipids (cyan) are more concentrated in SDD than elsewhere. White arrow indicates peripapillary SDD next to the optic nerve head. Scale bars apply to entire column: A, B, 200 µm; C, 100 µm. See also Figure 4.

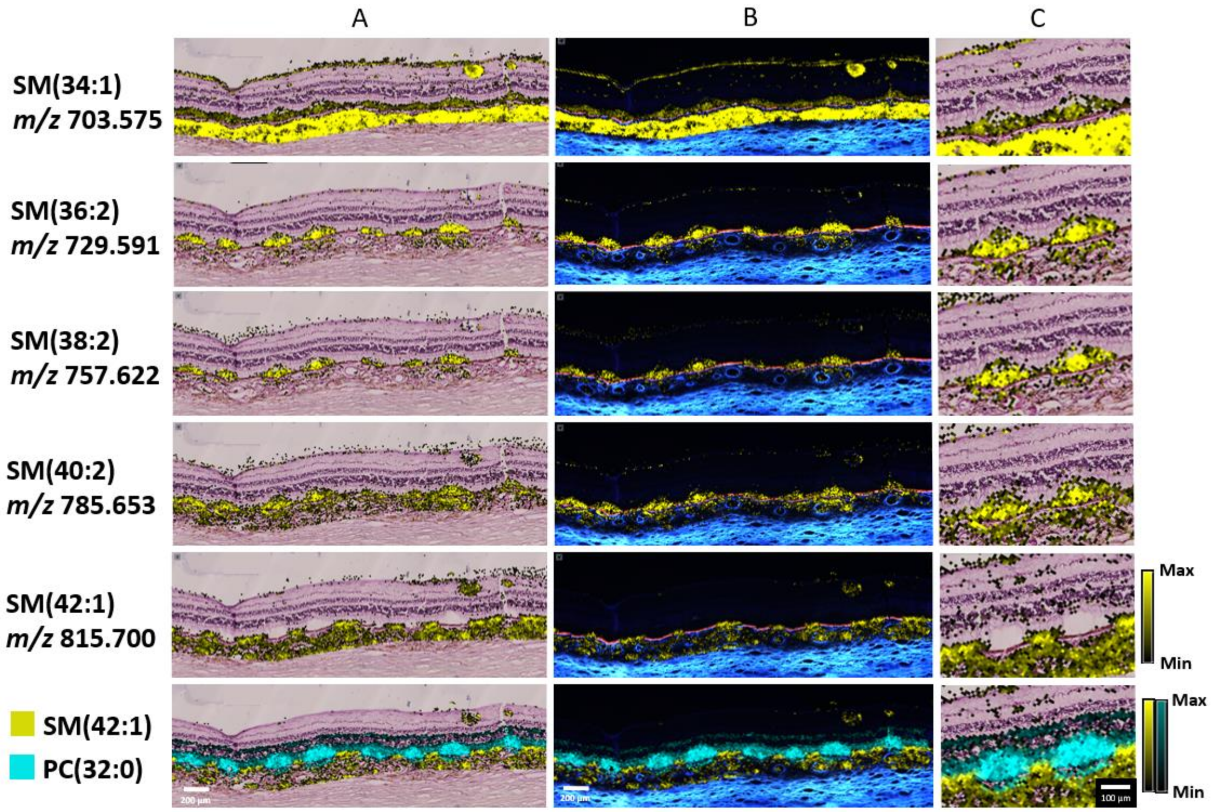

Figure S6. IMS of SM and PC lipids, Donor 1, positive ion mode.
Overlays of IMS images with H&E (A, C) and AF (B) images show that signals for SM (yellow) and PC (cyan) lipids are more concentrated in SDD than elsewhere. Scale bars apply to entire column: A, B, 200 µm; C, 100 µm. See also Figure 4.

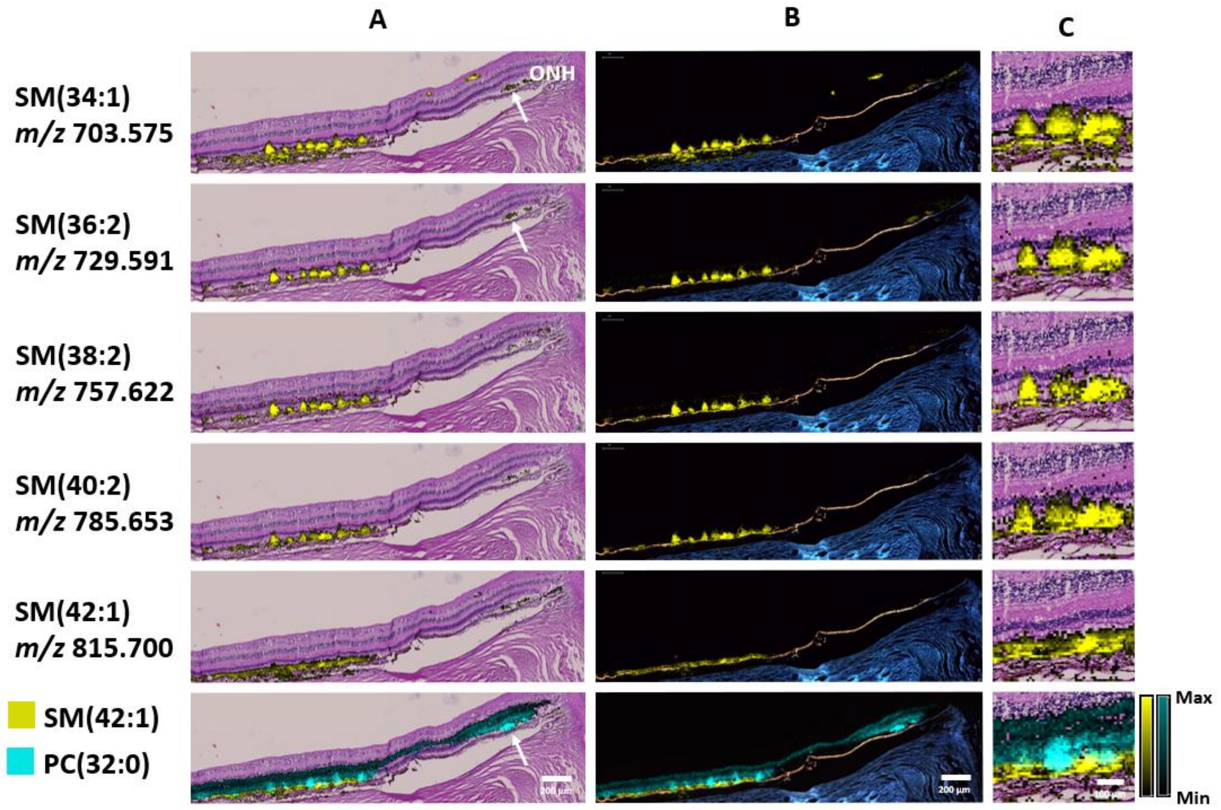

Figure S7. IMS of SM and PC lipids, Donor 2, positive ion mode.
Overlays of IMS images with H&E (A, C) and AF (B) images show that signals for SM (yellow) and PC (cyan) lipids are more intense in SDD than elsewhere. White arrow indicates peripapillary SDD directly next to the optic nerve head (right edge of image). Scale bars apply to entire column: A, B, 200 µm; C, 100 µm. See also Figure 4.

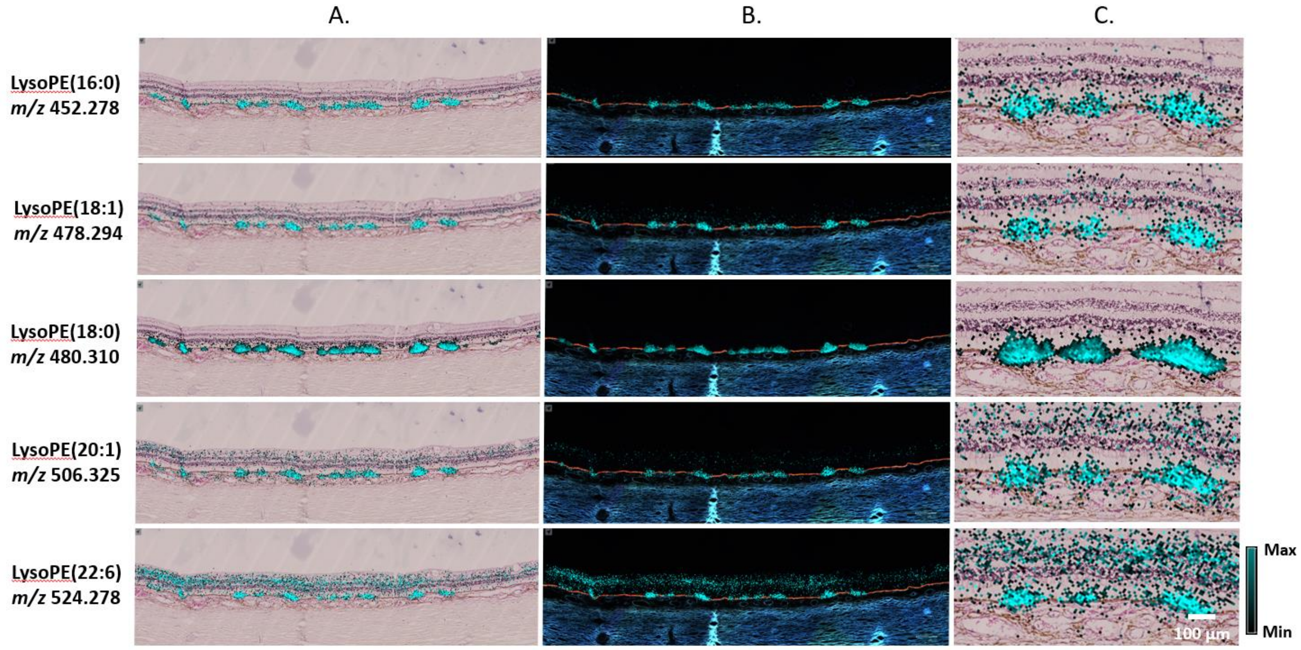

Figure S8. IMS of LysoPE lipids, Donor 1, negative ion mode.
Overlays of IMS images with H&E (A, C) and AF (B) images show that signals for LysoPE lipids (cyan) are more intense in SDD than elsewhere. Scale bars apply to entire column: A, B, 200 µm; C, 100 µm. See also Figure 5.

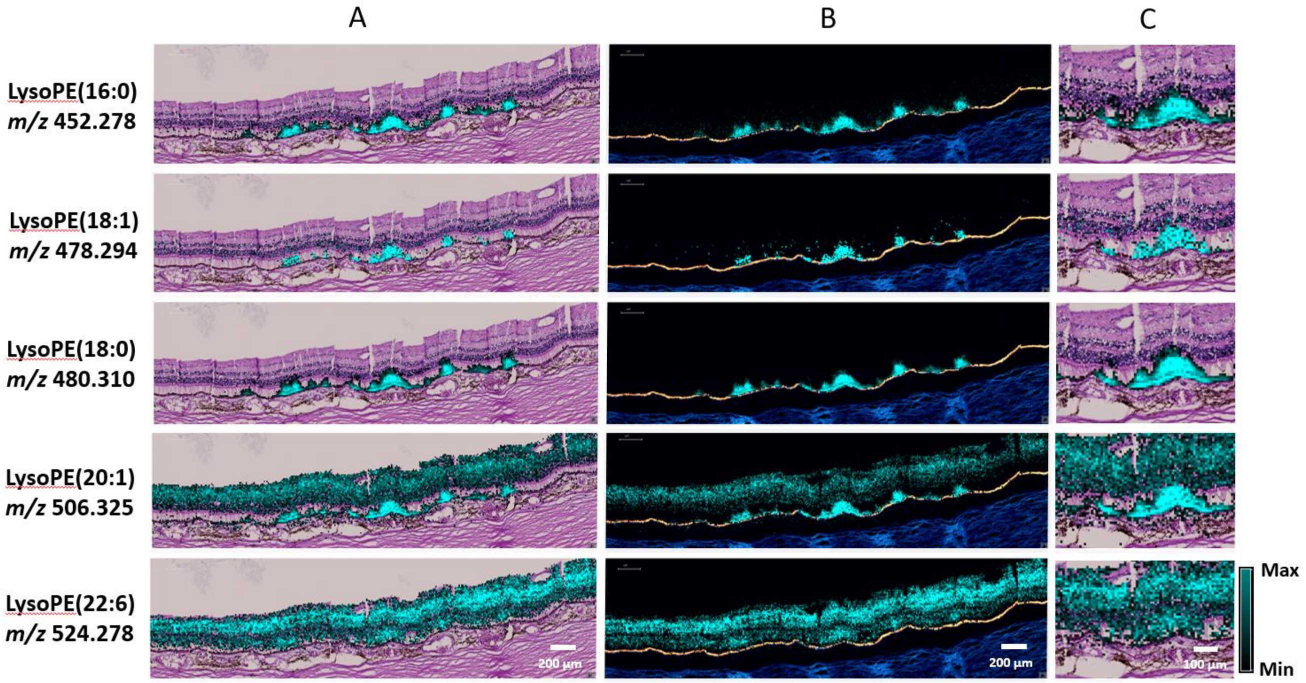

Figure S9. IMS of LysoPE lipids, Donor 2, negative ion mode.
Overlays of IMS images with H&E (A,C) and AF (B) images show that signals for LysoPE lipids (cyan) are more intense in SDD than elsewhere. Scale bars apply to entire column: A, B, 200 µm; C, 100 µm. See also Figure 5.

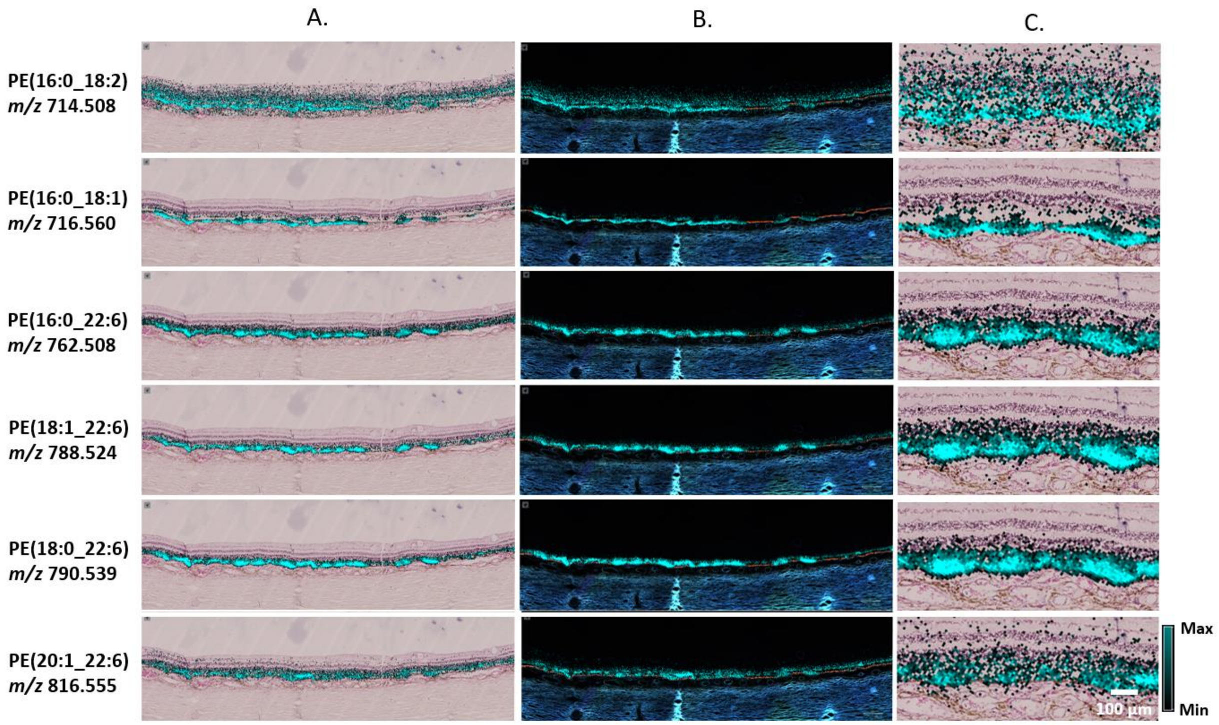

Figure S10. IMS of PE lipids, Donor 1, negative ion mode.
Overlays of IMS images with H&E (A,C) and AF (B) images show that signals for PE lipids (cyan) are more intense in SDD than elsewhere. Scale bars apply to entire column: A, B, 200 µm; C, 100 µm. See also Figure 5.

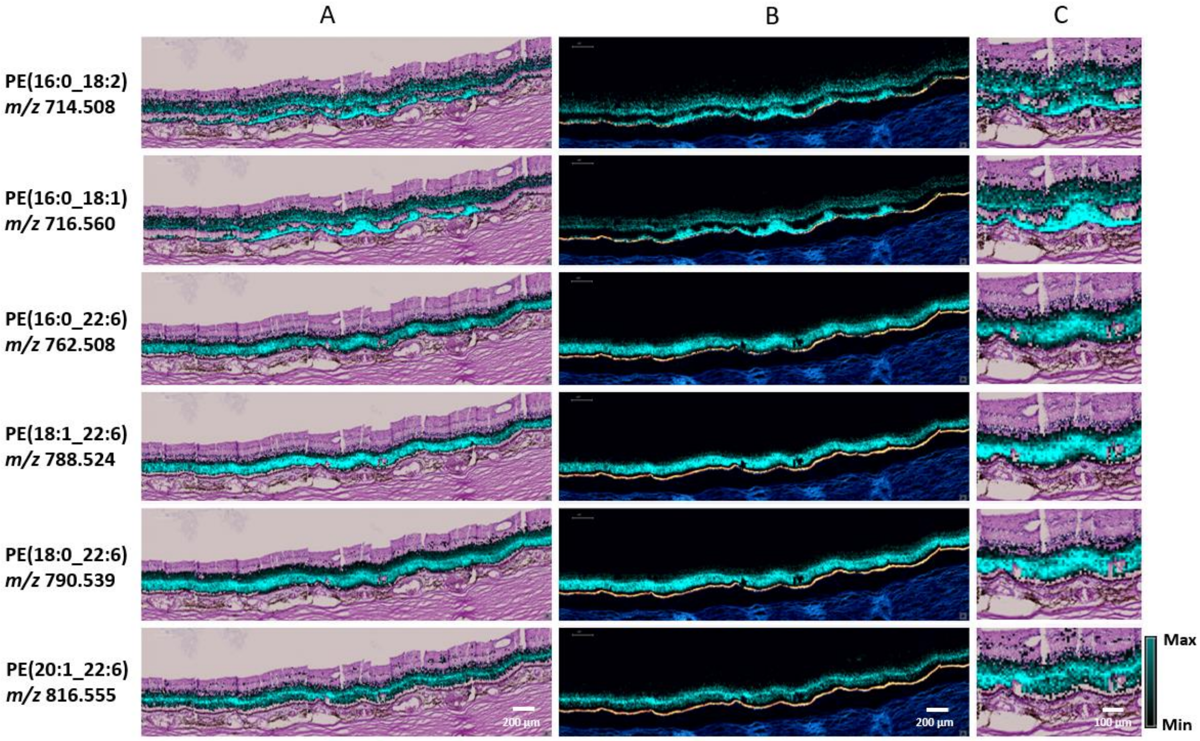

Figure S11. IMS of PE lipids, Donor 2, negative ion mode.
Overlays of IMS images with H&E (A, C) and AF (B) images show that signals for PE lipids (cyan) are not obviously concentrated in SDD. Scale bars apply to entire column: A, B, 200 µm; C, 100 µm. See also Figure 5.

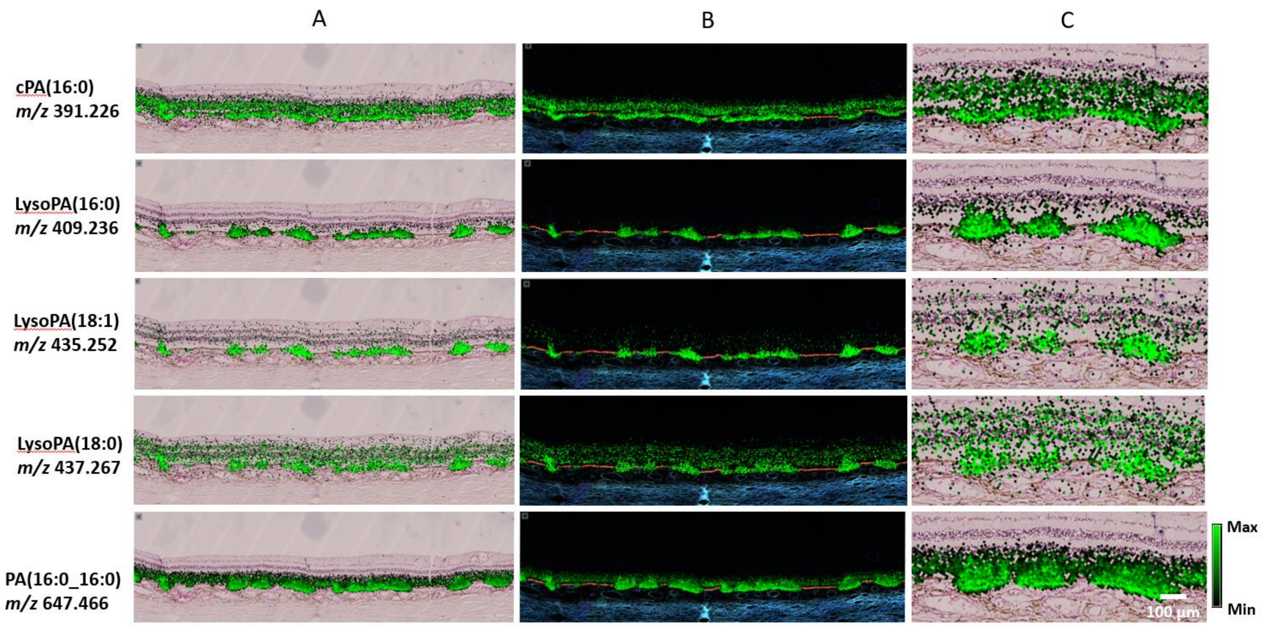

Figure S12. IMS of LysoPA and PA lipids, Donor 1, negative ion mode.
Overlays of IMS images with H&E (A,C) and AF (B) images show that signals for cPA, LysoPA and PA lipids (green) are more concentrated in SDD than elsewhere. Scale bars apply to entire column: A, B, 200 µm; C, 100 µm. See also Figure 5.

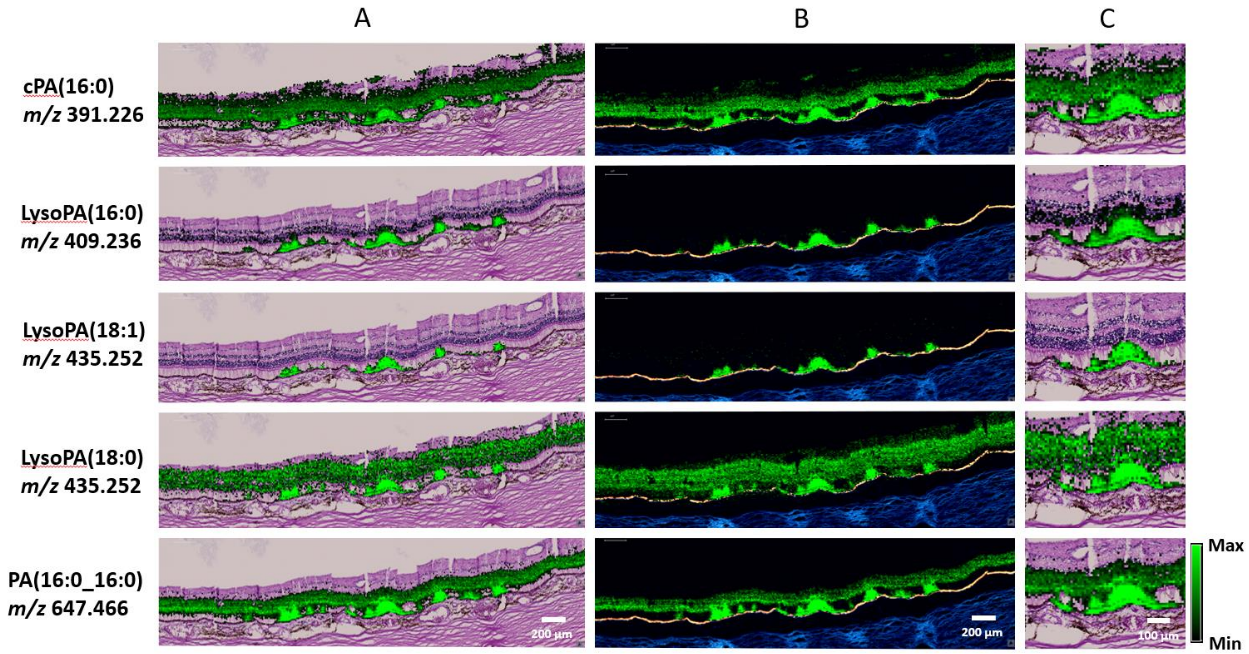

Figure S13. IMS of LysoPA and PA lipids, Donor 2, negative ion mode.
Overlays of IMS images with H&E (A,C) and AF (B) images show that signals for cPA, LysoPA, and PA lipids (green) are more intense in SDD than elsewhere. Scale bars apply to entire column: A,B, 200 µm; C, 100 µm. See also Figure 5.

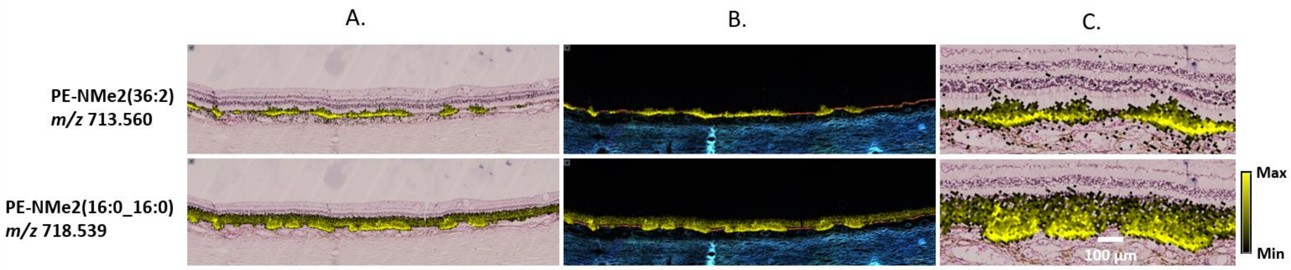

Figure S14. IMS of PE-NMe2 lipids, Donor 1, negative ion mode.
Overlays of IMS images with H&E (A,C) and AF (B) images show that signals for PE-NMe2 lipids (yellow) are more intense in SDD than elsewhere. Scale bars apply to entire column: A, B, 200 µm; C, 100 µm. See also Figure 5.

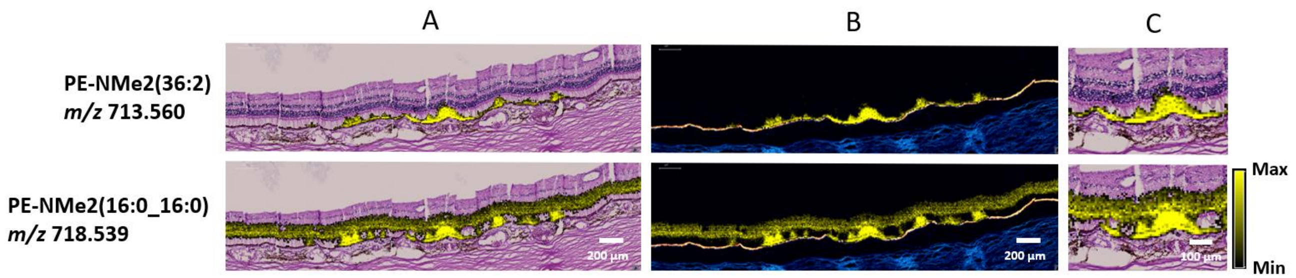

Figure S15. IMS of PE-NMe2 lipids, Donor 2, negative ion mode.
Overlays of IMS images with H&E (A, C) and AF (B) images show that signals for PE-NMe2 lipids (yellow) are more intense in SDD than elsewhere. Scale bars apply to entire column: A, B, 200 µm; C, 100 µm. See also Figure 5.

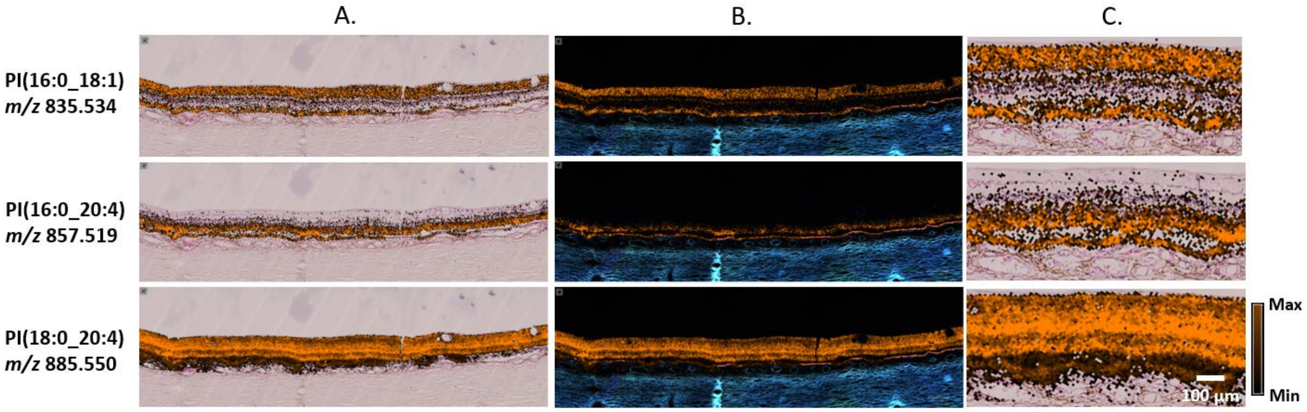

Figure S16. IMS of PI lipids, Donor 1, negative ion mode.
Overlays of IMS images with H&E (A, C) and AF (B) images show that signals for PI lipids (orange) are not obviously more intense in SDD than elsewhere. Scale bars apply to entire column: A, B, 200 µm; C, 100 µm. See also Figure 5.

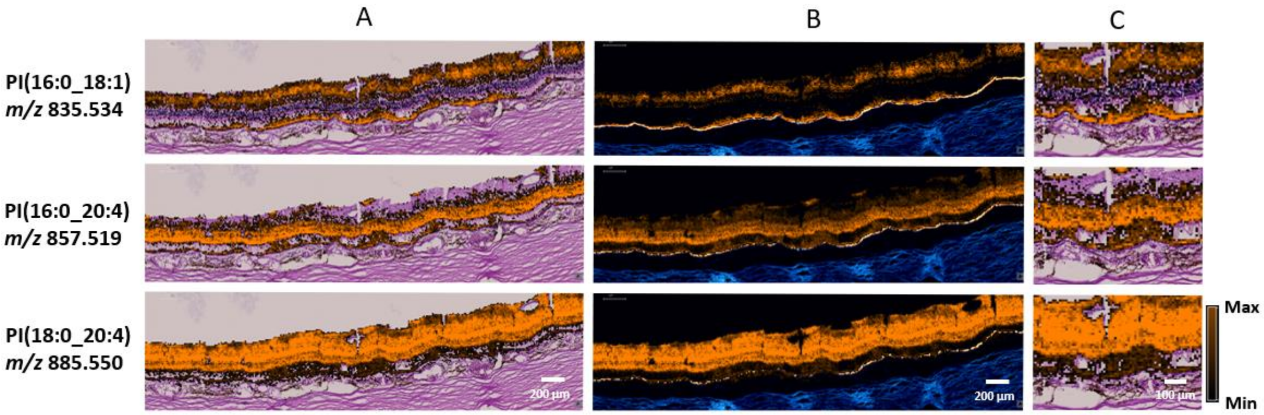

Figure S17. IMS of PI lipids, Donor 2, negative ion mode.
Overlays of IMS images with H&E (A, C) and AF (B) images show that signals for PI lipids (orange) are sparsely localized in SDD. Scale bars apply to entire column: A, B, 200 µm; C, 100 µm. See also Figure 5.

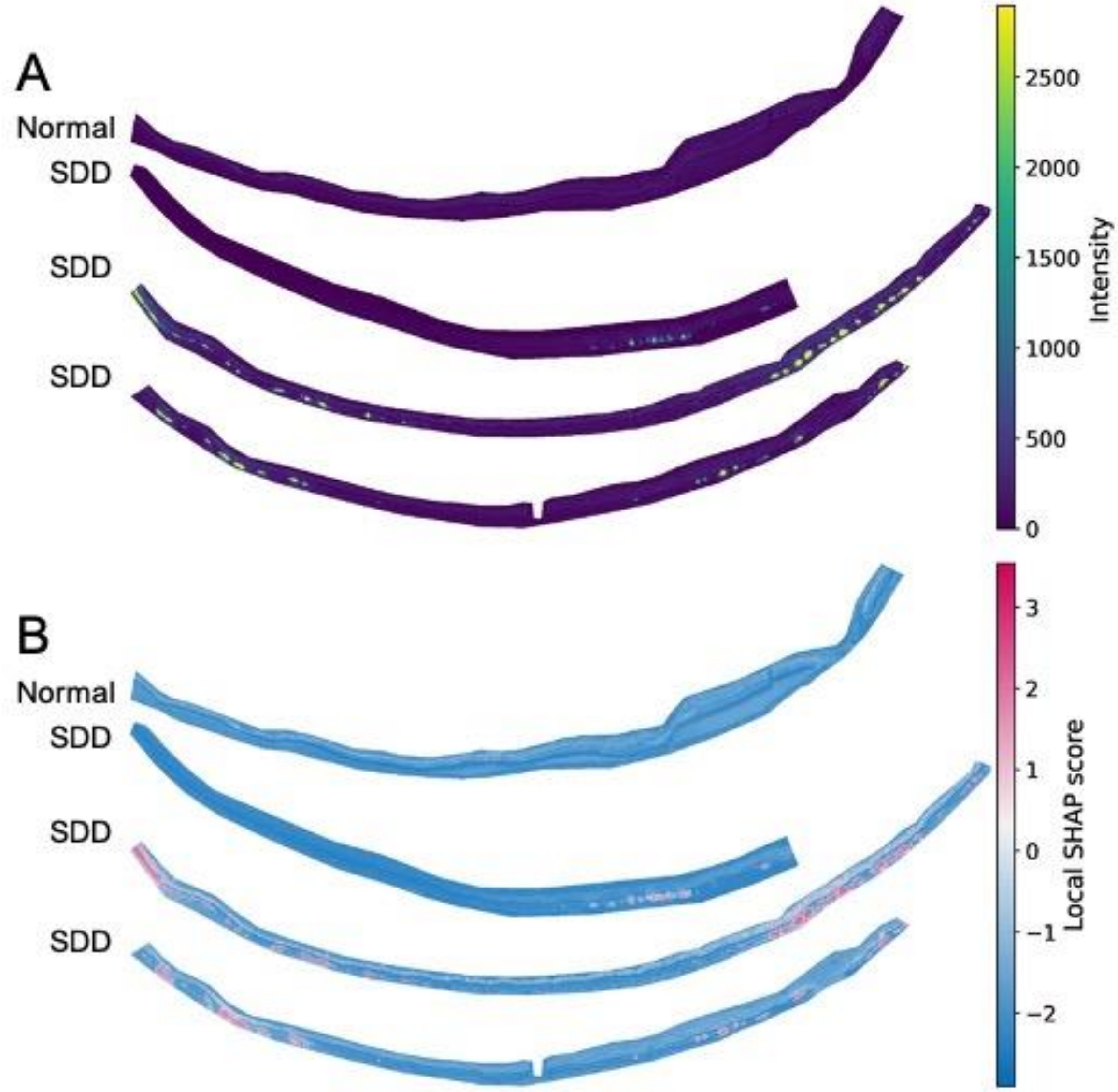

Figure S18. IMS images and SHAP maps of m/z 1277.897.

An example of a positively correlating marker candidate for SDD (in positive ion mode) that was not previously recognized or identified. (A) IMS images of m/z 1277.897 showing an increased intensity in SSD compared to non-SDD tissue. (B) SHAP maps (reporting localized SHAP importance) of m/z 1277.897 show the tissue areas (red) where this positive candidate marker improves the recognition of SDD tissue.

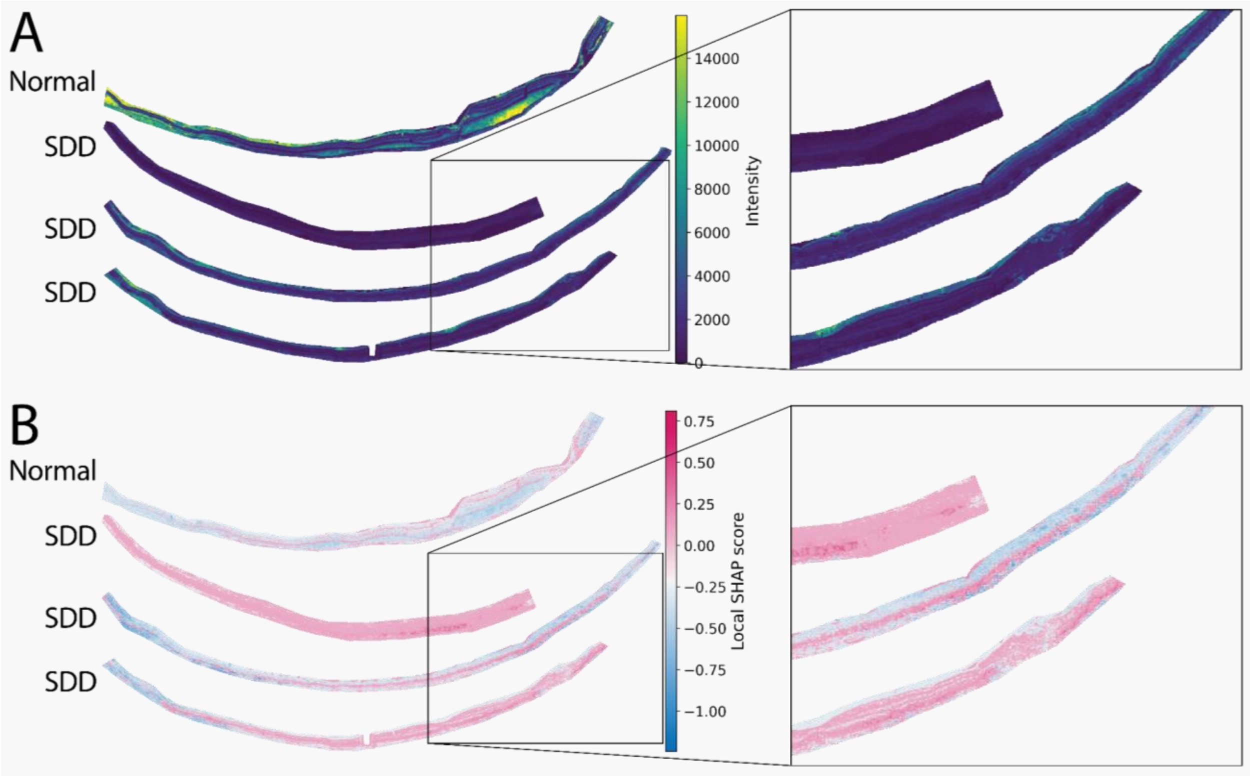

Figure S19. IMS images and SHAP maps of m/z 672.422.

An example of a negatively correlating marker candidate for SDD (in positive ion mode). Ion images of m/z 672.422 showing no signal in SDD compared to non-SDD tissue. (B) SHAP maps (reporting localized SHAP importance) show the tissue areas (red) where this negative candidate marker (m/z 672.422) improves the recognition of SDD tissue.

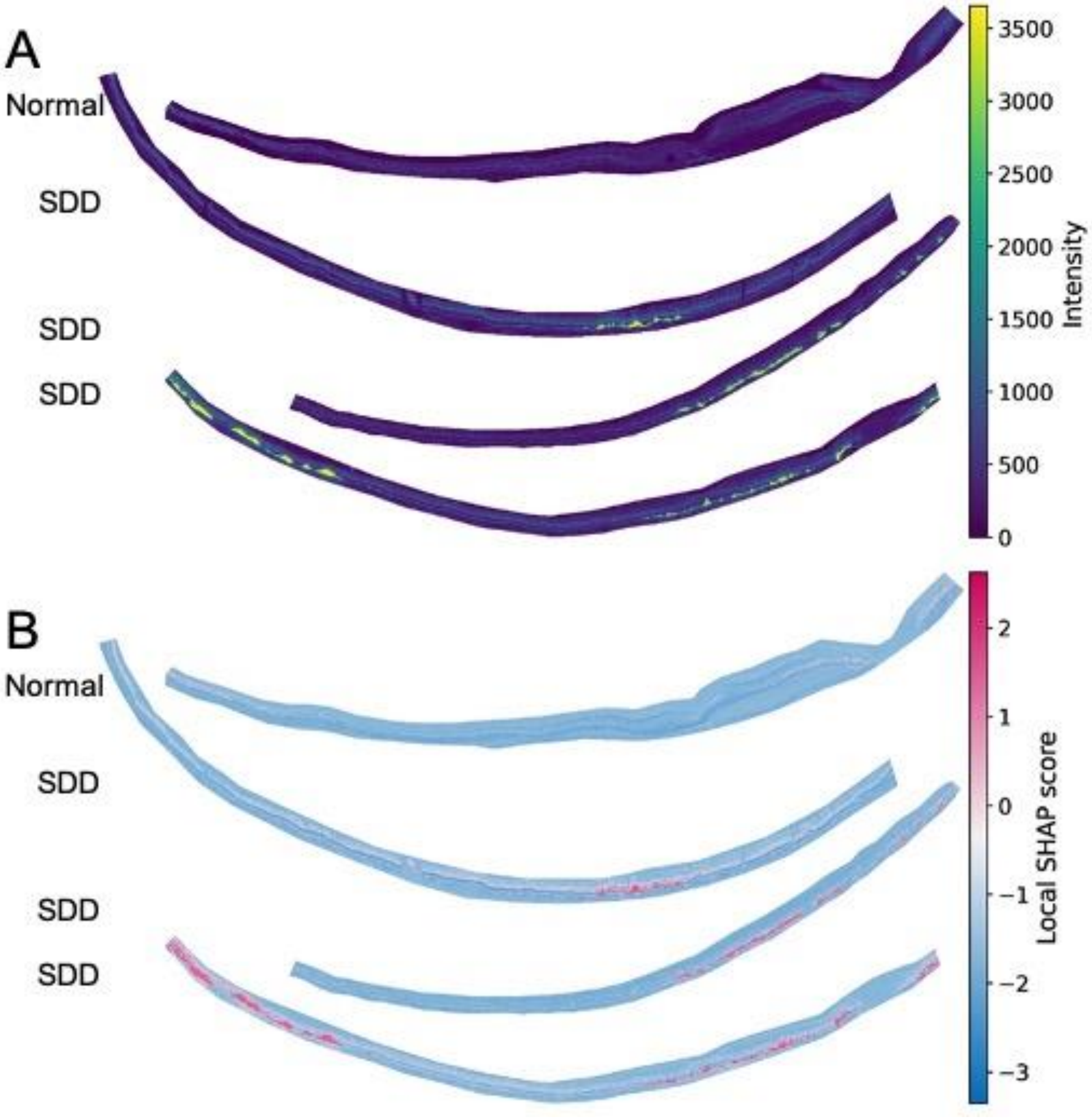

Figure S20. IMS images and SHAP maps of m/z 741.592.
An example of a positively correlating marker candidate for SDD (in negative ion mode) that was not previously recognized or identified. (A) Ion images showing an increased intensity of m/z 741.592 in SSD compared to non-SDD tissue. (B) SHAP maps (reporting localized SHAP importance) show the tissue areas (red) where this positive candidate marker improves the recognition of SDD tissue.

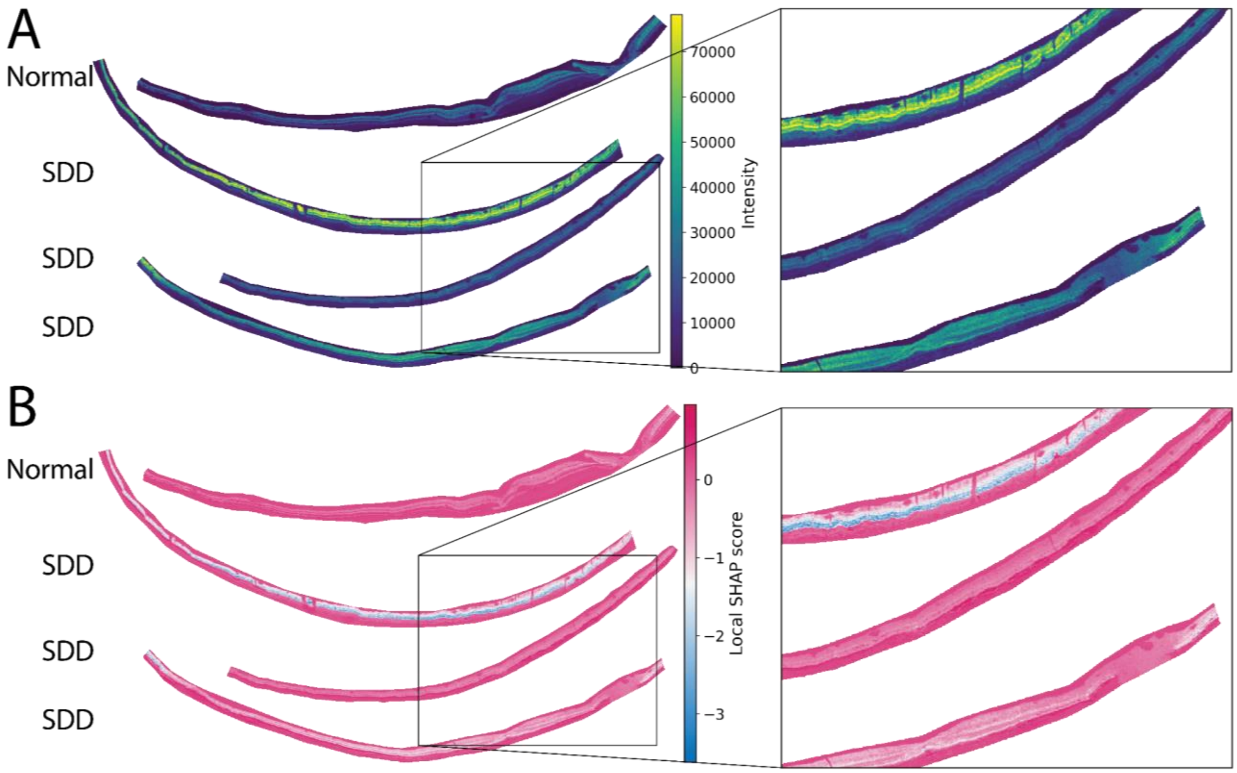

Figure S21. IMS images and SHAP maps of m/z 885.549.
An example of a negatively correlating marker candidate for SDD (in negative ion mode). (A) Ion images showing no signal for m/z 885.549 in SSD(B) SHAP maps (reporting localized SHAP importance) show the tissue areas (red) where this negative candidate marker (m/z 885.549) improves the recognition of SDD tissue.

Table S1: Donor eyes used in figures

Age	Sex	D-P Time (Hours)	Diagnosis (OCT, histologic)	Use	Figures	Relevant medical history
91	M	3.5	Early AMD	MALDI -IMS; LC-MS/MS	Figs. 4-7. Figs. S2, S4, S6, S8, S10, S12, S14, S16, S18-21.	Chronic heart failure; coronary artery disease; cardiac ablation
87	F	1.7	Early AMD	MALDI -IMS	Figs. 6-7, S3, S5, S7, S9, S11, S13, S15, S17, S18-21.	Past myocardial infarct; chronic obstructive pulmonary disease; hypertension; coronary artery disease
97	F	3.1	Early AMD	PASH	Fig. 2	Atrial fibrillation; myocardial infarct; pacemaker; renal failure
76	F	2.3	Early AMD	TEM	Fig. 3D-E,G-I	Renal carcinoma
88	M	4.5	Early AMD	TEM	Fig. 3A-C	Hypothyroidism, Parkinson disease, gastroesophageal reflux disease
-	-	-	Early AMD	TEM	Fig. 3F*	-
90	F	5.5	Atrophic AMD	LC-MS/MS	-	Not available
84	M	4.1	Unremarkable	IHC	Fig. 8A1-A4	Atrial fibrillation; coronary artery disease; hyperlipidemia; hypertension; myocardial infarction; cardiac allograft vasculopathy; fistula-in-ano
80	F	4.8	Unremarkable	IHC	Fig. 8B1-B4	Hypertension; atrial fibrillation; gastroesophageal reflux disease

Notes: AMD, age-related macular degeneration; IHC, immunohistochemical staining; LC-MS/MS, *Liquid Chromatography with tandem mass spectrometry*; SDD, subretinal drusenoid deposits; MALDI-MS, matrix-assisted laser desorption/ionization imaging mass spectrometry; PASH, periodic acid Schiff hematoxylin staining; TEM, transmission electron microscopy. * Clinically assessed by J.P. and S. H. Sarks; electron microscopic image from M.C. Killingsworth. No other information available.

Table S2. Tandem mass spectral fragmentation patterns, positive ion mode. LC-MS/S and MALDI IMS results showing lipid assignment, molecular ion mass, fragment ion masses, and mass accuracy of lipids observed in human retina with SDD by. Exp m/z, experimental mass-to-charge ratio; Th m/z, theoretical mass to charge ratio; ppm, parts per million; MF, molecular formula.

Lipid	Exp m/z	ID	Th m/z	ppm error	MF
**LysoPC(18:3)**	518.3237	LC-MS/MS [M+H]+	518.3241	0.8	C45H78O13P-
	184.0736	Phosphocholine loss	184.0739	1.6	C5H15NO5P+
	518.3218	MALDI [M+H]+	518.3241	4.3	C45H78O13P-
**LysoPC(18:1)**	522.3568	LC-MS/MS [M+H]+	522.3554	2.7	C26H53NO7P+
	184.0735	Phosphocholine loss	184.0739	2.2	C5H15NO5P+
	522.3554	MALDI [M+H]+	522.3554	2.7	C26H53NO7P+
**LysoPC(18:0)**	524.3726	LC-MS/MS [M+H]+	524.3711	2.9	C26H55NO7P+
	184.0736	Phosphocholine loss	184.0739	1.6	C5H15NO5P+
	524.3710	MALDI [M+H]+	524.3711	2.9	C26H55NO7P+
**LysoPC(20:4)**	544.3411	LC-MS/MS [M+H]+	544.3398	2.4	C28H51NO7P+
	184.0736	Phosphocholine loss	184.0739	1.6	C5H15NO5P+
	544.3382	MALDI [M+H]+	544.3398	2.4	C28H51NO7P+
**LysoPC(20:3)**	546.3577	LC-MS/MS [M+H]+	546.3554	4.2	C28H53NO7P+
	184.0735	Phosphocholine loss	184.0739	2.2	C5H15NO5P+
	546.3531	MALDI [M+H]+	546.3554	4.2	C28H53NO7P+
**PC(32:3)**	728.5239	LC-MS/MS [M+H]+	728.5225	1.9	C40H75NO8P+
	184.0736	Phosphocholine loss	184.0739	1.6	C5H15NO5P+
	728.5202	MALDI [M+H]+	728.5225	1.9	C40H75NO8P+
**SM (36:2)**	729.5922	LC-MS/MS [M+H]+	729.5905	2.3	C41H82N2O6P+
	184.0736	Phosphocholine loss	184.0739	1.6	C5H15NO5P+
	729.5902	MALDI [M+Na]+	729.5905	2.4	C41H81N2NaO6P+
**PC(32:1)**	754.5369	LC-MS/MS [M+H]+	754.5349	2.7	C42H77NO8P+
	184.0736	Phosphocholine loss	184.0739	1.6	C5H15NO5P+
	754.5369	MALDI [M+H]+	754.5349	1.7	C42H77NO8P+
**PC(32:0)**	756.5514	LC-MS/MS [M+H]+	756.5504	1.3	C42H79NO8P+
	184.0736	Phosphocholine loss	184.0739	1.6	C5H15NO5P+
	756.5513	MALDI [M+H]+	756.5504	1.3	C42H79NO8P+
**SM(38:2)**	757.6237	LC-MS/MS [M+H]+	757.6218	2.5	C43H86N2O6P+
	184.0736	Phosphocholine loss	184.0739	1.6	C5H15NO5P+
	757.6220	MALDI [M+Na]+	757.6218	2.5	C43H85N2NaO6P+
**PC(O-34:0)**	748.6233	LC-MS/MS [M+H]+	748.6215	2.4	C41H83NO8P+
	184.0736	Phosphocholine loss	184.0739	1.6	C5H15NO5P+
	748.6191	MALDI [M+H]+	748.6215	2.4	C41H83NO8P+
**SM(42:1)**	815.7018	LC-MS/MS [M+H]+	815.7000	2.2	C47H96N2O6P+
	184.0736	Phosphocholine loss	184.0739	1.6	C5H15NO5P+
	815.6992	MALDI [M+H]+	815.7000	2.2	C47H96N2O6P+
**SM(34:1)**	703.5760	LC-MS/MS [M+H]+	703.5748	1.7	C39H80N2O6P+
	184.0736	Phosphocholine loss	184.0739	1.6	C5H15NO5P+
	703.5749	MALDI [M+H]+	703.5748	1.7	C39H80N2O6P+
**SM(40:2)**	785.6553	LC-MS/MS [M+H]+	785.6531	2.8	C45H90N2O6P+
	184.0736	Phosphocholine loss	184.0739	1.6	C5H15NO5P+
	785.6529	MALDI [M+Na]+	785.6531	2.8	C45H89N2NaO6P+

Table S3 Tandem mass spectral fragmentation pattern, negative ion mode.
LC-MS/S and MALDI IMS results showing lipid assignment, molecular ion mass, fragment ion masses, and mass accuracy of lipids observed in human retina with SDD. Exp m/z, experimental mass/charge; Th m/z, theoretical mass to charge ratio; ppm, parts per million; MF, molecular formula.

Lipid	Exp m/z	Identification	Th m/z	ppm error	MF
LysoPA(16:0)	409.2354	LC-MS/MS [M-H]-	409.2361	1.7	C19H38O7P-
	255.2323	sn1 RCOO- glycerol-3-phosphate ion	255.2330	2.7	C16H31O2-
	171.0060	Loss of sn1 acyl chain as ketone (RCH=C=O) from [M-H]-	171.0064	2.3	C3H8O6P-
	152.9957	Neutral loss of sn1 RCOOH group from [M-H]-	152.9958	0.7	C3H6O5P-
	96.9695	H2PO4- ion (from phosphate)	96.9696	1.0	H2PO4-
	409.2321	MALDI [M-H]-	409.2361	0.3	C19H38O7P-
LysoPA(18:1)	435.2508	LC-MS/MS [M-H]-	435.2517	2.1	C21H40O7P-
	281.2480	sn1 RCOO- ion	281.2486	2.1	C18H33O2-
	171.0060	Loss of sn1 acyl chain as ketene (RCH=C=O) from [M-H]-	171.0064	2.3	C3H8O6P-
	152.9956	Neutral loss of sn1 RCOOH group from [M-H]-	152.9958	1.3	C3H6O5P-
	96.9694	H2PO4- ion (from phosphate)	96.9691	3.1	H2PO4-
	435.2487	MALDI [M-H]-	435.2517	0.3	C21H40O7P-
LysoPA (16:0_16:0)	647.4647	LC-MS/MS [M-H]-	647.4657	1.5	C35H68O8P-
	255.2322	sn2 RCOO- ion/ sn1 RCOO- ion	255.233	3.1	C16H31O2-
	152.9955	Glycerol-3-phosphate ion with loss of H2O	152.9958	2.0	C3H6O5P-
	96.9695	H2PO4- ion (from phosphate)	96.9696	1.0	H2PO4-
	647.4657	MALDI [M-H]-	647.4657	0.0	C35H68O8P-
CyclicPA(16:0)	391.2251	LC-MS/MS [M-H]-	391.2255	1.0	C19H36O6P-
	255.2325	sn1 RCOO- ion	255.2324	0.4	C16H31O2-
	283.2638	Loss of sn1 acyl chain as RCH=C=O-C-O-CH2-CH3	283.2637	0.4	C18H35O2-
	391.2229	MALDI [M-H]-	391.2255	0.1	C19H36O6P-
LysoPA(18:0)	437.2665	LC-MS/MS [M-H]-	437.2674	2.1	C21H42O7P-
	152.9956	Neutral loss of sn1 RCOOH group from [M-H]-	152.9958	1.3	C3H6O5P-
	96.9693	H2PO4- ion (from phosphate)	96.9696	3.1	H2PO4-
	437.2672	MALDI [M-H]-	437.2674	0.4	C21H42O7P-
LysoPE(18:1)	478.2927	LC-MS/MS [M-H]-	478.2939	2.5	C23H45NO7P-
	281.2480	sn1 RCOO- ion	281.2486	2.1	C18H33O2-
	214.0481	Loss of sn1 acyl chain as ketene (RCH=C=O) from [M-H]-	214.0486	2.3	C5H13NO6P-
	196.0376	Neutral loss of sn1 RCOOH group from [M-H]-	196.038	2.0	C5H11NO5P-
	152.9957	Glycerol-3-phosphate ion with loss of H2O	152.9958	0.7	C3H6O5P-
	140.0118	Ethanolamine phosphate ion	140.0118	0.0	C2H7NO4P-
	96.9697	H2PO4- ion (from phosphate)	96.9696	1.0	H2PO4-
	478.2937	MALDI [M-H]-	281.2486	0.4	C23H45NO7P-
LysoPE(18:0)	480.3085	LC-MS/MS [M-H]-	480.3096	2.3	C23H47NO7P-
	283.2637	sn1 RCOO- ion	283.2643	2.1	C18H35O2-
	214.0480	Loss of sn1 acyl chain as ketene (RCH=C=O) from [M-H]-	214.0486	2.8	C5H13NO6P-
	196.0374	Neutral loss of sn1 RCOOH group from [M-H]-	196.038	3.1	C5H11NO5P-
	152.9958	Glycerol-3-phosphate ion with loss of H2O	152.9958	0.0	C3H6O5P-

Lipid	Exp m/z	Identification	Th m/z	ppm error	MF
	140.0116	Ethanolamine phosphate ion	140.0118	1.4	C2H7NO4P-
	96.9697	H2PO4- ion (from phosphate)	96.9696	1.0	H2PO4-
	480.3094	MALDI [M-H]-	480.3096	0.3	C23H47NO7P-
**LysoPE(20:1)**	506.3240	LC-MS/MS [M-H]-	506.3252	2.4	C25H49NO7P-
	309.2795	sn1 RCOO- ion	309.2799	1.3	C20H37O2-
	214.0483	Loss of sn1 acyl chain as ketene (RCH=C=O) from [M-H]-	214.0486	1.4	C5H13NO6P-
	196.0380	Neutral loss of sn1 RCOOH group from [M-H]-	196.038	0.0	C5H11NO5P-
	152.9956	Glycerol-3-phosphate ion with loss of H2O	152.9958	1.3	C3H6O5P-
	140.0116	Ethanolamine phosphate ion	140.0118	1.4	C2H7NO4P-
	506.3251	MALDI [M-H]-	506.3252	0.2	C25H49NO7P-
**LysoPE(22:6)**	524.2769	LC-MS/MS [M-H]-	524.2783	2.7	C27H43NO7P-
	327.2324	sn1 RCOO- ion	327.233	1.8	C22H31O2-
	283.2425	Loss of CO2 from sn1 RCOO- ion (PUFA)	283.2431	2.1	C21H31-
	214.0481	Loss of sn1 acyl chain as ketene (RCH=C=O) from [M-H]-	214.0486	2.3	C5H13NO6P-
	196.0377	Neutral loss of sn1 RCOOH group from [M-H]-	196.038	1.5	C5H11NO5P-
	152.9955	Glycerol-3-phosphate ion with loss of H2O	152.9958	2.0	C3H6O5P-
	140.0115	Ethanolamine phosphate ion	140.0118	2.1	C2H7NO4P-
	78.9593	PO3- ion (from phosphate)	78.9591	2.5	PO3-
	524.2783	MALDI [M-H]-	524.2783	0.1	C27H43NO7P-
**LysoPE(20:4)**	500.2772	LC-MS/MS [M-H]-	500.2783	2.2	C25H43NO7P-
	303.2324	sn1 RCOO- ion	303.233	1.9	C20H31O2-
	259.2423	Loss of CO2 from sn1 RCOO- ion (PUFA)	259.2431	3.0	C19H31-
	214.0482	Loss of sn1 acyl chain as ketene (RCH=C=O) from [M-H]-	214.0486	1.8	C5H13NO6P-
	196.0380	Neutral loss of sn1 RCOOH group from [M-H]-	196.038	0.0	C5H11NO5P-
	152.9956	Glycerol-3-phosphate ion with loss of H2O	152.9958	1.3	C3H6O5P-
	140.0117	Ethanolamine phosphate ion	140.0118	0.7	C2H7NO4P-
	78.9583	PO3- ion (from phosphate)	78.9591	2.5	PO3-
	500.2780	MALDI [M-H]-	500.2783	0.6	C25H43NO7P-
**LysoPE(16:0)**	452.2773	LC-MS/MS [M-H]-	452.2783	2.2	C21H43NO7P-
	255.2325	sn1 RCOO- ion	255.233	2.0	C16H31O2-
	214.0483	Loss of sn1 acyl chain as ketene (RCH=C=O) from [M-H]-	214.0486	1.4	C5H13NO6P-
	196.0376	Neutral loss of sn1 RCOOH group from [M-H]-	196.038	2.0	C5H11NO5P-
	152.9954	Glycerol-3-phosphate ion with loss of H2O	152.9958	2.6	C3H6O5P-
	140.0117	Ethanolamine phosphate ion	140.0118	0.7	C2H7NO4P-
	78.9593	PO3- ion (from phosphate)	78.9591	2.5	PO3-
	452.2788	MALDI [M-H]-	452.2783	1.0	C21H43NO7P-
**PE(16:0_18:2)**	714.5063	LC-MS/MS [M-H]-	714.5079	2.2	C39H73NO8P-
	279.2325	sn2 RCOO- ion	279.233	1.8	C18H31O2-
	255.2325	sn1 RCOO- ion	255.233	2.0	C16H31O2-
	140.0115	Ethanolamine phosphate ion	140.0118	2.1	C2H7NO4P-
	78.9593	PO3- ion (from phosphate)	78.9591	2.5	PO3-
	714.5068	MALDI [M-H]-	714.5079	1.5	C39H73NO8P-
	716.5208	LC-MS/MS [M-H]-	716.5236	3.9	C39H75NO8P-
	452.2766	Loss of sn2 acyl chain as ketene (RCH=C=O) from [M-H]-	452.2783	3.8	C21H43NO7P-

Lipid	Exp m/z	Identification	Th m/z	ppm error	MF
**PE(16:0_18:1)**	281.2482	sn2 RCOO- ion	281.2486	1.4	C18H33O2-
	255.2326	sn1 RCOO- ion	255.233	1.6	C16H31O2-
	140.0117	Ethanolamine phosphate ion	140.0118	0.7	C2H7NO4P-
	716.5240	MALDI [M-H]-	716.5236	0.5	C39H75NO8P-
**PE-NMe2(36:2)**	713.5575	LC-MS/MS [M-H]-	713.5597	3.1	C40H78N2O6P-
	447.2991	Loss of sn2 acyl chain as ketene (RCH=C=O)	447.2987	0.9	C22H44N2O5P-
	168.0429	Phosphocholine with loss of CH3	168.0431	1.2	C4H11NO4P-
	78.9594	PO3- ion (from phosphate)	78.9591	3.8	PO3-
	713.5602	MALDI [M-H]-	713.5597	0.7	C40H78N2O6P-
**PE-NMe2 (16:0_16:0)**	718.5374	LC-MS/MS [M-H]-	718.5392	2.5	C39H77NO8P-
	480.3088	Loss of sn2 acyl chain as ketene (RCH=C=O)	480.309	0.4	C23H47NO7P-
	255.2325	sn1 RCOO- ion	255.233	2.0	C16H32O2-
	224.0689	Glycerophosphocholine with loss of CH3 and H2O	224.0693	1.8	C7H16NO5P-
	168.0429	Phosphocholine with loss of CH3	168.0431	1.2	C4H11NO4P-
	78.9594	PO3- ion (from phosphate)	78.9591	3.8	PO3-
	718.5376	MALDI [M-H]-	718.5392	2.2	C39H77NO8P-
**PE(16:0_22:6)**	762.5065	LC-MS/MS [M-H]-	762.5079	1.8	C43H73NO8P-
	452.2775	Loss of sn2 acyl chain as ketene (RCH=C=O) from [M-H]	452.2783	1.8	C21H31O2-
	327.2324	sn2 RCOO- ion	327.233	1.8	C22H31O2-
	283.2427	Loss of CO2 from sn2 RCOO- ion (PUFA)	283.2431	1.4	C21H31-
	255.2326	sn1 RCOO- ion	255.233	1.6	C16H31O2-
	140.0117	Ethanolamine phosphate ion	140.0118	0.7	C2H7NO4P-
	78.9594	PO3- ion (from phosphate)	78.9591	3.8	PO3-
	762.5086	MALDI [M-H]-	762.5079	1.0	C43H73NO8P-
**PE(18:1_22:6)**	788.5219	LC-MS/MS [M-H]-	788.5236	2.2	C45H75NO8P-
	478.2931	Loss of sn1 acyl chain as ketene (RCH=C=O) from [M-H]-	478.2939	1.7	C23H45NO7P-
	327.2325	sn1 RCOO- ion	327.233	1.5	C22H31O2-
	281.2481	sn2 RCOO- ion	281.2486	1.8	C18H33O2-
	140.0117	Ethanolamine phosphate ion	140.0118	0.7	C2H7NO4P-
	78.9594	PO3- ion (from phosphate)	78.9591	3.8	PO3-
	788.5247	MALDI [M-H]-	788.5236	8.5	C45H75NO8P-
**PE(20:1_22:6)**	816.5547	LC-MS/MS [M-H]-	816.5549	0.2	C47H79NO8P-
	506.3252	Loss of sn2 acyl chain as ketene (RCH=C=O) from [M-H]-	506.3252	0.0	C25H49NO7P-
	327.2323	sn2 RCOO- ion	327.233	2.1	C22H31O2-
	309.2796	sn1 RCOO- ion	309.2799	1.0	C20H37O2-
	140.0115	Ethanolamine phosphate ion	140.0118	2.1	C2H7NO4P-
	78.9594	PO3- ion (from phosphate)	78.9591	3.8	PO3-
	816.5547	MALDI [M-H]-	816.5549	0.2	C47H79NO8P-
**PE(18:0_22:6)**	790.5383	LC-MS/MS [M-H]-	790.5392	1.1	C45H77NO8P-
	480.3089	Loss of sn2 acyl chain as ketene (RCH=C=O) from [M-H]-	480.3096	1.5	C23H47NO7P-
	327.2325	sn2 RCOO- ion	327.233	1.5	C22H31O2-
	283.2638	sn1 RCOO- ion	283.2643	1.8	C18H35O2-
	140.0118	Ethanolamine phosphate ion	140.0118	0.0	C2H7NO4P-
	78.9594	PO3- ion (from phosphate)	78.9591	3.8	PO3-
	790.5392	MALDI [M-H]-	790.5392	0.0	C45H77NO8P-
**PE(18:1_22:6)**	788.5217	LC-MS/MS [M-H]-	788.5236	2.4	C45H75NO8P-
	478.2928	Loss of sn2 acyl chain as ketene (RCH=C=O) from [M-H]-	478.2939	2.3	C23H45NO7P-
	327.2323	sn2 RCOO- ion	327.233	2.1	C22H31O2-
	281.2479	sn1 RCOO- ion	281.2486	2.5	C18H33O2
	140.0117	Ethanolamine phosphate ion	140.0118	0.7	C2H7NO4P-

Lipid	Exp m/z	Identification	Th m/z	ppm error	MF
	788.5247	MALDI [M-H]-	788.5236	1.4	C45H75NO8P-
PI(16:0_20:4)	857.5179	LC-MS/MS [M-H]-	857.5186	0.8	C45H78O13P-
	571.2880	Loss of sn2 acyl chain as ketene (RCH=C=O) from [M-H]-	571.2889	1.6	C25H48O12P-
	409.2355	Loss of sn2 acyl chain as ketene (RCH=C=O) and inositol from [M-H]-	409.2361	1.5	C19H38O7P-
	303.2324	sn2 RCOO- ion	303.233	2.0	C20H31)2-
	255.2324	sn1 RCOO- ion	255.233	2.4	C16H31O2-
	241.0112	Inositol phosphate ion - H2O	241.0119	2.9	C6H10O8P-
	152.9956	Glycerol-3-phosphate ion with loss of H2O	152.9958	1.3	C3H6O5P-
	96.9696	H2PO4- ion (from phosphate)	96.9696	0.0	H2PO4-
	857.5183	MALDI [M-H]-	857.5186	0.3	C45H78O13P-
PI(18:0_20:4)	885.5488	LC-MS/MS [M-H]-	885.5499	1.2	C47H82O13P-
	581.3091	Neutral loss of sn2 RCOOH group from [M-H]-	581.3096	0.9	C27H50O11P-
	303.2324	sn2 RCOO- ion	303.233	2.0	C20H31O2-
	283.2638	sn1 RCOO- ion	283.2643	1.8	C18H35O2-
	259.2427	Loss of CO2 from sn2 RCOO- ion (PUFA)	259.2431	1.5	C19H31-
	241.0112	Inositol phosphate ion - H2O	241.0119	2.9	C6H10O8P-
	152.9956	Glycerol-3-phosphate ion with loss of H2O	152.9958	1.3	C3H6O5P-
	96.9692	H2PO4- ion (from phosphate)	96.9696	4.1	H2PO4-
	885.5466	MALDI [M-H]-	885.5499	3.7	C47H82O13P-
PI(16:0_18:1)	835.5335	LC-MS/MS [M-H]-	835.5342	0.8	C43H80O13P-
	281.2478	sn2 RCOO- ion	281.2486	2.8	C18H33O2-
	255.2325	sn1 RCOO- ion	255.233	2.0	C16H31O2-
	241.0113	Inositol phosphate ion - H2O	241.0119	2.5	C6H10O8P-
	152.9956	Glycerol-3-phosphate ion with loss of H2O	152.9958	1.3	C3H6O5P-
	96.9694	H2PO4- ion (from phosphate)	96.9696	2.1	H2PO4-
	835.5310	MALDI [M-H]-	835.5342	3.8	C43H80O13P-

Supplemental Materials and Methods

Human donor eyes

Whole eyes were obtained from deceased human donors by Advancing Sight Network (Birmingham, AL) as part of ongoing studies on age-related macular degeneration (AMD) that are approved by institutional review at University of Alabama at Birmingham (protocol # N170213002), where tissues were collected. Acceptable donors were ≥ 80 years of age, white, non-diabetic, and received ≤6 hours death-to-preservation. Ophthalmic history was not available. In this demographic, AMD is prevalent, and eyes were screened for AMD presence and staging using *ex vivo* multimodal imaging including optical coherence tomography (OCT) (1), a widely used clinical diagnostic imaging technology. As described (1), subretinal drusenoid deposits (SDD) were

recognized as intermittent reflectivities with a dentate appearance internal to the RPE-basal lamina -Bruch's membrane band on OCT. Although SDD are not yet part of any clinical AMD classification system, they confer risk for advanced AMD (2,3), and they may appear in other acquired or inherited retinal diseases. In this age-range with evidence of other AMD characteristics (drusen and dysmorphic RPE), we assume that these eyes are AMD. Two eyes of two donors (Donor 1 and Donor 2) meeting these criteria were processed for MALDI-IMS (details in **Supplementary Table S1**). Eyes from a previous series of eyes harvested the same way were used for diagnostic stains (see below).

Tissue Section Collection

As described (4), tissues were preserved by overnight immersion in 4% paraformaldehyde in 0.1 M phosphate buffer at 4°C then prepared for cryosections. In brief, the cornea was removed, and the iris incised radially to allow fixative to infiltrate the globe. Globes were immersed in phosphate buffered 4% paraformaldehyde for 24 h, then in 1% paraformaldehyde until processed. Before embedding in carboxymethylcellulose (CMC) prior to cryosectioning, the posterior pole was trimmed to a 14-mm-wide belt of retina, choroid, and sclera containing the optic nerve head and fovea and extending anteriorly to the pigmented ora serrata at the edge of the ciliary body.

Tissue mounted indium tin oxide (ITO) slides (Delta Technologies ETC) were vacuum sealed with oxygen absorbing packets and transported to Vanderbilt University on dry ice and stored in a -80˚C freezer. Before analysis, slides were brought to room temperature and dried in a vacuum desiccator for a minimum of 30 minutes.

MALDI IMS analysis

MALDI matrices, 1,5-diaminonaphthalene (DAN) for negative ion mode and 2,5-dihydroxyacetophenone (DHA) for positive ion mode, were applied to tissue sections using a custom designed sublimation device. MALDI IMS data were acquired with a 10 μm pixel size with a 10 μm pitch in full scan mode using a timsTOF Pro MALDI imaging

platform in QTOF mode (Bruker Daltonik, Bremen, Germany). Data were acquired with 250 laser shots per pixel and within a mass range of m/z 300−2000. The mass spectrometer was calibrated with red phosphorus prior to data acquisition (5). Following IMS data acquisition, tissue sections were stained with hematoxylin and eosin (H&E) to permit spatial registration of IMS signals to tissue layers. Sections were imaged by bright-field microscopy and, both pre- and post-IMS acquisition, by fluorescence microscopy for autofluorescence (AF), which is especially intense for the RPE layer. For H&E staining, the matrix was removed with a light methanol rinse. Slides were immersed in 95 and 70% ethanol for 30 seconds before a 20 second dip in H_2O, slides were then placed in hematoxylin for 3 minutes then rinsed by sequential dips (~5) in clean H_2O. Tissues were subsequently dehydrated by immersing slides in 70 and 95% ethanol for 30 seconds, followed by 1 minute in eosin. Excess eosin was removed by immersing the slides in 95 and 100% ethanol for 30 seconds each before placing the slide in xylene for 3 minutes and mounting the coverslip with (Cytoseal XYL, ThermoFisher Scientific). Data were initially processed using SCiLS Lab MVS (Version 2023a Pro) to find ions of interest before exporting the data to an advanced image registration workflow using *IMS MicroLink* and *wsireg*, incorporating both AF and bright-field microscopy images in the Vitessce visualization tool (6-9). Images were thresholded on an individual basis to give the best contrast to and to allow for clear localizations to be determined. IMS data were exported for accurate registration as TIFF images.

MALDI IMS data processing

Data were exported from the Bruker timsTOF file format (.d) to a custom binary format for ease of access and improved performance. Each pixel/frame contains between 10^4-10^5 centroid peaks that cover the entire acquired m/z range, which can be used to reconstitute a pseudo-profile mass spectrum using Bruker's timsTOF SDK software (v2.21). The IMS datasets were m/z-aligned using internal peaks, specifically, 6 peaks that appeared in at least 50% of the pixels, using the *msalign* library (v0.2.0)(10). This step corrects spectral misalignments (drift along the m/z domain), resulting in improved overlap between spectral features (peaks) across the group of IMS datasets.

Subsequently, the mass axis of each dataset was calibrated using a minimum of 4 calibrant species to correct for mass errors, where each dataset was corrected to approximately ±1 ppm precision. After m/z alignment and calibration, an average mass spectrum for dataset was computed across all pixels of that IMS dataset, specific to each ionization mode. The aim was feature detection in the average mass spectra from all IMS datasets in each ionization mode; however, despite the alignment and calibration, the mass axes are still slightly different for each dataset. To overcome this, we resample each mass spectrum in each dataset to a single common mass axis with slightly rougher spectral sampling (approximately 1.5 ppm; spacing between adjacent mass bins) and only then calculate an average mass spectrum. For each ionization mode, the average mass spectrum was peak-picked and a total of 983 and 890 peaks were detected in the positive and negative ionization mode, respectively. Note that isotopic peaks were not removed in the classification workflow. Following these m/z-axis pre-processing steps, we computed normalization correction factors for each of the pixels in the datasets to assure comparability of ion intensities across IMS datasets. To this end, we used a total ion current (TIC) strategy, modified for robustness, where only intensity data between the 5 and 95% percentiles is summed together to form the TIC (5-95% TIC). The intensity values that fall outside of this 5/95% percentile window were exempted from the normalization correction to reduce the impact of potential outlier peaks/features on normalization.

Supervised machine learning analysis & automated marker candidate discovery

To avoid subjective assessment and to ensure an exhaustive search for SDD-relevant marker candidates among the hundreds of ions mapped by IMS, we employed an interpretable supervised machine learning approach to compare SDD to regions of uninvolved retina and to automatically discern candidate markers. A complete description of the approach can be found in Tideman et al.(11). Briefly, we first trained a multivariate classification model that assigns IMS pixels to different biological classes, i.e., “SDD tissue area” versus “non-SDD tissue area”, based on the mass spectrum of each pixel. Subsequently, an interpretable machine learning approach named Shapley additive explanations (SHAP) was used to examine how the trained classification model

made decisions to label IMS pixels as ‘SDD’ or ‘non-SDD’. The SHAP method allowed all IMS-measured molecular species to be automatically ranked in decreasing order of relevance to the model’s recognition of SDD. The top-ranked ion species on this list are marker candidates for SDD, either by positive or negative correlation to SDD.

Data labelling/mask creation

To make the classification model capture IMS variation that is relevant to retina tissue with SDD, we labelled IMS pixels as either *SDD* or *background*. The positive class (pixels labeled as *SDD*) was manually assigned in napari (v0.4.2) (12), based on low-dimensional latent patterns extracted by non-negative matrix factorization of the IMS data. Our initial labeling was subsequently compared to features seen by histological staining and corrected if needed. The negative class (*background*-labeled pixels) was automatically created sampling non-positive class pixels, effectively excluding SDD pixels from being in the *background* class. Since the number of background-labeled pixels is much larger than the number of *SDD*-labeled pixels, we performed auto-balancing of the training data. The balancing is performed by randomly sampling examples from the negative class pixels such that the number of pixels in the positive and negative class is roughly equal (~5,700 pixels/class in negative ion mode and ~3,650 pixels/class in positive ion mode).

Building an SDD classification model

We employed eXtreme Gradient Boosting (XGBoost)(13) to learn a tree-based model that can classify mass spectra as either *SDD* or *background*. The model was trained with a 67%/33% train/test split. The classification workflow was carried out using the scikit-learn (v1.0.2) and XGBoost (v1.5.2) libraries in Python (v3.8.11). The classifier models achieved good SDD-recognition performance for negative and positive ion mode (balanced accuracy, 0.9854, 0.9821; precision 0.9911, 0.9831; recall 0.9798, 0.9815). These numbers indicate that, for both ionization modes, a model was found that can recognize SDD well given the IMS-detected ions provided. Since these models capture a strong connection between the IMS data and SDD, exploring their decision process by SHAP can yield molecular species relevant to SDD.

Lipid extraction and nLC-MS/MS analysis

Sections of two human donor retinas with SDD were processed for lipid extraction for nLC-MS/MS analysis. Twelve-µm-thick paraformaldehyde-fixed tissue sections were extracted. Under a dissecting microscope, sclera was removed with a number 10 scalpel blade. From the remaining retina-choroid tissue, regions with SDD were scraped from the glass slide and placed in a 1.5-mL HPLC glass vial. One milliliter of MMC extraction solvent (1.3:1:1, methanol: methyl tert-butyl ether: chloroform) was spiked with 10 µL of lipid standard mixture (SPLASH® LIPIDOMIX® Mass Spec Standard, Avanti Polar Lipids, Alabaster, AL, USA), vortexed for 60 s and centrifuged at 3000 rpm for 10 min (4,14). The supernatant was transferred to a separate HPLC vial and was evaporated to dryness. The sample was then reconstituted with 10 µL n-butanol: isopropyl aclcohol:H_20 (8:23:69) with 5 mM phosphoric acid, and 1 µL was injected each for positive and negative ion mode analysis by nLC–MS/MS.

In-house packed reverse phase columns (20 cm x 75 µm) were packed with 1.9 µm BEH C-18 material and nLC-MS/MS analysis was performed using an EASY nLC 1000 (Thermo Scientific). A gradient mobile phase comprised of solvent A (10 mM ammonium formate in 40:60 water: acetonitrile by volume with 0.1% formic acid) and Solvent B (10 mM ammonium formate in 90:10 isopropanol: acetonitrile by volume with 0.1% formic acid) was used. A 300 nL/min flow rate was used for 120 minutes, and the column compartment was heated to 60 ºC. The gradient elution profile was as follows: 1-30% B (0-12 min), 30%–51% B (12-16 min), 51% B (16-20 min), 51%–61% B (20–40min), 61%–71% B (40-60 min), 71%–99% B (60–80 min), 99% B (80–100 min), 99%–1% B (100–110 min), 1% B (110–120 min) (15,16).

HPLC eluate flowed into an ESI source, and ions were analyzed using a Q Exactive HF instrument (Thermo Scientific, San Jose, CA, USA). Data were acquired in both full (MS1) and data dependent MS2 (ddMS2) scan modes using positive and negative modes separately. The full scan mode had a mass resolution of 60,000, a mass range of m/z 200–2000, and a maximum trap fill time of 100 ms. ddMS2 data were acquired at

15,000 resolutions, with a maximum trap fill time of 160 ms. The isolation window of selected MS1 ions was ±1.4 m/z with a normalized collision energy (NCE) of 20 and 25. LC–MS/MS data were acquired using Xcalibur version 4.0.

Lipid identification

Both positive and negative MALDI timsTOF raw data files were imported into LipostarMSI software (Molecular Horizon srl, Perugia, IT) (17) for processing, image co-registration, statistical analysis, and lipid identification. Data were recalibrated using the m/z 885.5499 signal in negative ion mode and m/z 725.5456, m/z 756.5514, m/z 760.5851, m/z 782.5670, and m/z 784.5851 in positive ion mode. nLC–MS/MS data acquired from serial section homogenates were imported into LipostarMSI to identify lipids in the SDD regions of interest (ROIs). ROIs were determined based on histology and on supervised data analysis (PCA and bisecting k-means segmentation (18). A co-localization algorithm was then applied to isolate the ion markers statistically correlated to the pathological ROIs, further confirmed by visual inspection of ion images (19). Molecular identification was automatically assigned by LipostarMSI based on accurate MS and MS/MS matching (≤10 ppm, possible adducts: +H, +Na, +K, -H, +Cl; dimers and oxidized species for GP and GL allowed) against the LIPIDMAPS Database (http://www.lipidmaps.org/ accessed on 6 January 2022). Only molecules that could be definitively identified by LC-MS/MS fragments were reported. The entire data analysis workflow was performed independently on two biological replicates.

PASH staining and light microscopy

For AMD diagnosis and comparison to *ex vivo* OCT, cryosections through the fovea and perifovea (recognized by Henle fiber layer) near those used for MALDI-IMS were stained with periodic acid Schiff hematoxylin (PASH, Poly Scientific R&D Corp., Bay Shore, NY, USA; #K047 kit). This stain highlights Bruch's membrane and sub-RPE deposits. Sections retained from a previous immunohistochemistry study (20) i.e., a third donor eye, was subject to the same characterization described above, with overall better morphologic preservation, were perused for good examples of SDD . Slides were dehydrated through 85%, 95%, 100% ethanol, and 100% xylene (Fisher, # X3S-4) for 5

minutes, twice at each concentration. All slides were cover-slipped with permanent medium (Permount, EMS, # 17986-01) and air-dried in a hood overnight.

To facilitate translation to clinical OCT imaging, which has signal in every pixel, bright field images were acquired for all stained slides. One stained section per slide was scanned with a 20X objective and a robotic microscope stage (Olympus VSI 120, CellSens; Olympus, Center Valley PA), scaled to tissue units. Sections were centered on the fovea or for non-foveal sections, where Henle fibers diverge, using a custom plugin for FIJI (https://imagej.nih.gov/ij/download.html). *Ex vivo* OCT B-scans and scanned whole sections were matched for major landmarks (e.g., overall tissue contour, foveal center, large vessels, individual pathologies). For details, some stained sections were scanned with a 60X oil immersion objective (numerical aperture = 1.4).
We used differential interference contrast (DIC) microscopy with 40X and 60X objectives to determine if material was visible in unstained dome- or flame-shaped areas lacking photoreceptor outer segments in the subretinal space. To compare our tissue sample to a clinical OCT staging system, we assessed SDD height relative to photoreceptors in cryosections stained for PASH in these and other similarly accessioned and processed AMD eyes (20-22). Stage 1 deposits impacted only outer segments, Stage 2 reached the inner segments, and Stage 3 deposits intruded on inner segments.

Transmission electron microscopy

To visualize the ultrastructure of SDD and membranes of cells surrounding the deposits, we prepared AMD tissue samples from prior studies for transmission electron microscopy. One block (Case 1, 76-year-old female) came from a histologic study of micro-dissected and pelleted drusen (23). Another block (Case 2, 88-year-old-male) came from macula-wide sections processed for the Project MACULA website (24). Both were preserved in 1% glutaraldehyde and 2.5% paraformaldehyde in phosphate buffer. To preserve extracellular lipids, samples were post-fixed with osmium tannic acid paraphenylenediamine (OTAP) (25,26). Tissue blocks from Cases 1-2 were embedded in epoxy resin (PolyBed 812, EMS, Hatfield PA) and re-sectioned for this study. Gold

sections (nominally 90 nm thick) were mounted on grids (Formvar Carbon Support Film on Specimen Grid, Electron Microscopy Sciences, Fort Washington, PA) and post-stained with mixed lanthanides (UranyLess, Electron Microscopy Sciences, Fort Washington, PA). TEM images were acquired at original magnifications of up to 2100x (Tecnai 120kv TEM, FEI, Hillsboro, OR; BioSprint 29 Megapixel CCD camera, AMT, Woburn, MA). An electron micrograph of clinically documented SDD (Case 3) was made available from the Sarks Archive (Sydney Australia; Data Use Agreement 2021STE02369); this eye had been post-fixed in 2% osmium.

Immunohistochemistry

Our finding of abundant lysolipids in SDD, and the finding that the monogenic inherited disorder phospholipase A2 group V (PLA2G5) retinopathy has an SDD phenotype(27,28), prompted us to confirm that the gene product localized to the retina. Cryosections from two normal donor eyes were processed for colorimetric immunohistochemical staining with rabbit polyclonal anti-human PLA2G5 (Fisher Scientific, Cat# PIPA584150, https://www.fishersci.com/shop/products/pla2g5 polyclonal-antibody-invitrogen-1/PIPA584150), following previously described methods(29). Briefly, after heat-induced antigen retrieval with unmasking solution (Vector Labs, Cat# H-3300) and 3 blocking steps ((BLOXALL, Vector labs, Cat# SP-6000), avidin/biotin blocking kit (Vector Labs, Cat# SP-2001) and 10% horse serum (Vector Labs, Cat# S2000-20) in 1x blocking solution (ThermoFisher, Cat# PI37525)), cryosections were incubated with PLA2G5 primary antibody (1: 50, ThermoFisher, Cat#PA584150), horse antirabbit biotinylated secondary antibody, ABC Complex solution (Vector Labs, Cat# PK-7200), and diaminobenzidine horseradish peroxidase substrate (Vector Labs, Cat# SK-4100) for the desired brown color. Stained sections on glass slides were scanned with a UPlanSApo 20x (20x/0.75, 00/0.17/FN26.5), 40x (40x/0.95, 00/0.11-0.23/FN26.5), and PlanApo N 60x (60x/1.42 Oil, 00/0.17/FN26.5) objectives and a robotic microscope stage (Olympus VS120, Olympus Japan).

Preparation of microscopy figures

Images for figures were assembled and adjusted for contrast, exposure, and sharpness to maximize the intensity histogram for contrast and white balance (Photoshop, CS6; Adobe Systems, San Jose, CA). To facilitate translation of results to clinical OCT, retinal layers were horizontally oriented with choroid down in all figures.

Supplemental Information References

1. Messinger JD, Brinkmann M, Kimble JA, Berlin A, Freund KB, Grossman GH, Ach T, Curcio CA. Ex vivo OCT-based multimodal imaging of human donor eyes for research in age-related macular degeneration. J Vis Exp (2023) doi: 10.3791/65240
2. Domalpally A, Agron E, Pak JW, Keenan TD, Ferris FL 3rd, Clemons TE, Chew EY. Prevalence, risk, and genetic association of reticular pseudodrusen in age-related macular degeneration: Age-Related Eye Disease Study 2 Report 21. Ophthalmology (2019) 126:1659-1666.
3. Tsai AS, Cheung N, Gan AT, Jaffe GJ, Sivaprasad S, Wong TY, Cheung CM. Retinal angiomatous proliferation. Surv Ophthalmol (2017) 62:462-492.
4. Kotnala A, Anderson DMG, Patterson NH, Cantrell LS, Messinger JD, Curcio CA, Schey KL. Tissue fixation effects on human retinal lipid analysis by MALDI imaging and LC-MS/MS technologies. J Mass Spectrom (2021) 56:e4798.
5. K. Sládková, J. Houska, J. Havel, Laser desorption ionization of red phosphorus clusters and their use for mass calibration in time-of-flight mass spectrometry. Rapid Commun Mass Spectrom (2009) 23:3114-3118.
6. Anderson DMG, Messinger JD, Patterson NH, Rivera ES, Kotnala A, Spraggins JM, Caprioli RM, Curcio CA, Schey KL. Lipid Landscape of the Human Retina and Supporting Tissues Revealed by High-Resolution Imaging Mass Spectrometry. J Am Soc Mass Spectrom (2020) 31:2426-2436.
7. Patterson NH, Tuck M, Van de Plas R, Caprioli RM, Advanced Registration and Analysis of MALDI Imaging Mass Spectrometry Measurements through Autofluorescence Microscopy. Anal Chem (2018) 90:12395-12403.
8. Keller MS, Gold I, McCallum C, Manz T, Kharchenko PV, Gehlenborg N. Vitessce: a framework for integrative visualization of multi-modal and spatially-resolved single-cell data. OSF Preprints (2021) doi:10.31219/osf.io/y8thv
9. Manz T, Gold I, Patterson NH, McCallum C, Keller MS, Herr BW 2nd, Börner K, Spraggins JM, Gehlenborg N. Viv: multiscale visualization of high-resolution multiplexed bioimaging data on the web. Nat Methods (2022)19:515-516.
10. Monchamp P, Andrade-Cetto L, Zhang JY, Henson R. "Signal processing methods for mass spectrometry" in G. Alterovitz and M.F. Ramoni, editors. Systems Bioinformatics: An Engineering Case-Based Approach. Artech House Publishers (2007) pp. 101-124.
11. Tideman LEM, Migas LG, Djambazova KV, Patterson NH, Caprioli RM, Spraggins JM, Vand de Plas R. Automated biomarker candidate discovery in imaging mass spectrometry data through spatially localized Shapley additive explanations. Analytica Chimica Acta (2021) 1177:338522.
12. Sofroniew N, Lambert T, Evans K, Nunez-Iglesias J, Bokota G, Winston P, Peña-Castellanos G, Yamauchi K, Bussonnier M, Doncila Pop D, Can Solak A, Liu Z, Wadhwa P, Burt A, Buckley G, Sweet A, Migas L, Hilsenstein V, Gaifas L, Bragantini J, Rodríguez-Guerra J, Muñoz H, Freeman J, Boone P, Lowe A, Gohlke C, Royer L, Pierré A, Har-Gil H, McGovern A. napari: a multi-dimensional image viewer for Python (v0.4.16rc7). (2022) Zenodo. https://doi.org/10.5281/zenodo.6597502
13. Chen T, Guestrin C. Xgboost: A scalable tree boosting system. Proceedings of the 22nd ACM SIGKDD International Conference on Knowledge Discovery and Data Mining; 2016 August 13-17; San Francisco, CA USA. pp. 785-794.

14. Pellegrino RM, Di Veroli A, Valeri A, Goracci L, Cruciani G. LC/MS lipid profiling from human serum: a new method for global lipid extraction. Anal Bioanal Chem (2014) 406:7937-7948.
15. Danne-Rasche N, Coman C, Ahrends R. Nano-LC/NSI MS Refines Lipidomics by Enhancing Lipid Coverage, Measurement Sensitivity, and Linear Dynamic Range. Anal Chem (2018) 90:8093-8101.
16. Vasilopoulou CG, Sulek K, Brunner AD, Meitei NS, Schweiger-Hufnagel U, Meyer SQW, Barsch A, Mann M, Meier F. Trapped ion mobility spectrometry and PASEF enable in-depth lipidomics from minimal sample amounts. Nat Commun (2020) 11:331.
17. Tortorella S, Tiberi P, Bowman AP, Claes BSR, Scupakova K, Heeren RMA, Ellis SR, Cruciani G. LipostarMSI: Comprehensive, Vendor-Neutral Software for Visualization, Data Analysis, and Automated Molecular Identification in Mass Spectrometry Imaging. J Am Soc Mass Spectrom (2020) 31:155-163.
18. Trede D, Schiffler S, Becker M, Wirtz S, Steinhorst K, Strhlow J, Aichler M, Kobarg JH, Oetjen J, Dyatlov A, Heldmann S, Walch A, Thiele H, Maass P, Alexandrov T. Exploring three-dimensional matrix-assisted laser desorption/ionization imaging mass spectrometry data: three-dimensional spatial segmentation of mouse kidney. Anal Chem (2012) 84:6079-6087.
19. McDonnell LA, van Remoortere A, van Zeijl RJ, Deelder AM. Mass spectrometry image correlation: quantifying colocalization. J Proteome Res (2008) 7:3619-3627.
20. Cao D, Leong B, Messinger JD, Kar D, Ach Y, Yannuzzi LA, Freund KB, Curcio CA . Hyperreflective Foci, Optical Coherence Tomography Progression Indicators in Age-Related Macular Degeneration, Include Transdifferentiated Retinal Pigment Epithelium. Invest Ophthalmol Vis Sci (2021) 62:34.
21. Chen L, Messigner JD, Zhang Y, Spaide RF, Freund KB, Curcio CA . Subretinal Drusenoid Deposit in Age-Related Macular Degeneration: Histologic Insights Into Initiation, Progression to Atrophy, and Imaging. Retina (2020) 40:618-631.
22. Zweifel SA, Spaide RF, Curcio CA, Malek G, Imamura Y. Reticular pseudodrusen are subretinal drusenoid deposits. Ophthalmology (2010) 117:303-312.e1.
23. Rudolf M, Clark ME, Chimento MF, Li C-M, Medeiros NE, Curcio CA. Prevalence and morphology of druse types in the macula and periphery of eyes with age-related maculopathy. Invest Ophthalmol Vis Sci (2008) 49:1200-1209.
24. Brinkmann M, Bacci T, Kar D, Messinger JD, Sloan KR, Chen L, Hamann T, Wiest M, Freund KB, Sweifel S, Curcio CA. Histology and Clinical Lifecycle of Acquired Vitelliform Lesion, a Pathway to Advanced Age-Related Macular Degeneration. Am J Ophthalmol (2022) 240:99-114.
25. Curcio CA, Millican CL, Bailey T, Kruth HS. Accumulation of cholesterol with age in human Bruch's membrane. Invest Ophthalmol Vis Sci (2001) 42:265-274.
26. Guyton JR, Klemp KF. Ultrastructural discrimination of lipid droplets and vesicles in atherosclerosis: value of osmium-thiocarbohydrazide-osmium and tannic acid-paraphenylenediamine techniques. J Histochem Cytochem (1988) 36:1319-1328.
27. Sergouniotis PI, Davidson AE, Mackay DS, Lenassi E, Li Z, Robson AG, Yang X, Kam JH, Isaacs TW, Holder GE, Jeffery G, Beck JA, Moore AT, Plagnol V, Webster AR. Biallelic mutations in PLA2G5, encoding group V phospholipase A2, cause benign fleck retina. Am J Hum Genet. 2011 Dec 9;89(6):782-91.

28. Khan KN, Mahroo OA, Khan RS, Mohamed MD, McKibbin M, Bird A, Michaelides M, Tufail A, Moore AT. Differentiating drusen: Drusen and drusen-like appearances associated with ageing, age-related macular degeneration, inherited eye disease and other pathological processes. Prog Retin Eye Res. 2016 Jul;53:70-106.
29. Cao D, Leong B, Messinger JD, Kar D, Ach T, Yannuzzi LA, Freund KB, Curcio CA. Hyperreflective Foci, Optical Coherence Tomography Progression Indicators in Age-Related Macular Degeneration, Include Transdifferentiated Retinal Pigment Epithelium. Invest Ophthalmol Vis Sci. 2021 Aug 2;62(10):34.
